# Enhancing Infotainment Services in Integrated Aerial–Ground Mobility Networks

**DOI:** 10.3390/s25133891

**Published:** 2025-06-22

**Authors:** Chenn-Jung Huang, Liang-Chun Chen, Yu-Sen Cheng, Ken-Wen Hu, Mei-En Jian

**Affiliations:** 1 Department of Computer Science & Information Engineering, National Dong Hwa University, Hualien 974301, Taiwan; 611321208@gms.ndhu.edu.tw (Y.-S.C.); 611221227@gms.ndhu.edu.tw (M.-E.J.); 2 Department of Information Management, National Taipei University of Nursing and Health Sciences, Taipei 108306, Taiwan; liangchun@ntunhs.edu.tw; 3 Fortra, Eden Prairie, MN 55344, USA; kaiwen.hu@fortra.com

**Keywords:** aerial–ground mobility, electric vehicle, resource management, infotainment services, 6G

## Abstract

The growing demand for bandwidth-intensive vehicular applications—particularly ultra-high-definition streaming and immersive panoramic video—is pushing current network infrastructures beyond their limits, especially in urban areas with severe congestion and degraded user experience. To address these challenges, we propose an aerial-assisted vehicular network architecture that integrates 6G base stations, distributed massive MIMO networks, visible light communication (VLC), and a heterogeneous aerial network of high-altitude platforms (HAPs) and drones. At its core is a context-aware dynamic bandwidth allocation algorithm that intelligently routes infotainment data through optimal aerial relays, bridging connectivity gaps in coverage-challenged areas. Simulation results show a 47% increase in average available bandwidth over conventional first-come-first-served schemes. Our system also satisfies the stringent latency and reliability requirements of emergency and live infotainment services, creating a sustainable ecosystem that enhances user experience, service delivery, and network efficiency. This work marks a key step toward enabling high-bandwidth, low-latency smart mobility in next-generation urban networks.

## 1. Introduction

The rapid development of smart transportation systems and autonomous driving technologies is transforming electric vehicles (EVs) into always-connected mobile hubs. Passengers increasingly demand seamless high-bandwidth services—such as 4K video streaming, immersive 360° video, and live video calls—which require ultra-stable internet connectivity. These requirements pose significant challenges in urban environments, where network congestion and fluctuating speeds are prevalent.

Current vehicular networks are often unable to satisfy the stringent quality-of-service (QoS) demands of real-time applications, including interactive video and remote medical support, which are highly sensitive to latency and interruptions. Although fifth-generation (5G) networks provide substantial improvements over previous generations, they still struggle to maintain reliable performance in dense urban areas during peak traffic hours. Emerging sixth-generation (6G) technologies, such as terahertz (THz) wireless communication and massive multiple-input multiple-output (mMIMO) antenna arrays, promise greater capacity and speed but remain insufficient for guaranteeing delay-critical services in high-mobility scenarios.

Prior research has investigated partial solutions, including drone-assisted signal enhancement, cloud-based network management, and edge computing. However, these approaches primarily address isolated aspects—such as coverage extension or computational offloading—without providing a comprehensive framework for seamless video delivery, dynamic bandwidth allocation, and multi-tier communication.

To address these challenges, this paper proposes a ground–air integrated network architecture that synergizes 6G base stations, roadside units (RSUs), high-speed visible light communication (VLC) links, high-altitude platforms (HAPs), and drones. The system features dynamic bandwidth allocation that prioritizes critical services (e.g., emergency video), mitigates congestion, and enhances network reliability. The key contributions include the following:

- A multi-layer hybrid network combining RSUs, drones, and HAPs interconnected via high-speed wireless links (VLC, mmWave, and THz) to provide robust connectivity for moving EVs.
- A dynamic, priority-based bandwidth allocation mechanism that adapts in real time to EV speed and service requirements, reserving capacity for high-priority tasks while efficiently managing lower-priority traffic.
- An aerial-assisted load balancing strategy whereby drones and HAPs function as mobile relays, redistributing unused bandwidth from low-demand to congested zones to support group streaming efficiently.
- A graceful video degradation scheme that anticipates network dead zones and preloads non-live content to prevent buffering interruptions.

Simulation results demonstrate that the proposed system can reduce congestion by increasing available bandwidth by up to 47% compared to state-of-the-art ground–air networks [1]. This improvement supports more stable and responsive video delivery, even during peak hours in dense urban environments, thus advancing the future of in-vehicle connectivity.

## 2. Related Work

The transformation of EVs into mobile entertainment hubs has significantly increased the demand for reliable, high-bandwidth connectivity. Passengers expect uninterrupted streaming of ultra-high-definition video, immersive virtual reality experiences, and real-time communication while traveling through urban areas where network performance is often unstable due to interference and congestion [2,3,4]. Although 5G cellular infrastructure represents a major advancement, it still faces challenges in maintaining consistent service quality in dense traffic environments and high-speed mobility scenarios, largely due to spectrum limitations and backbone network constraints [5,6]. Emerging 6G technologies leveraging extremely high-frequency radio waves and advanced antenna arrays show promise [7,8], yet their application to vehicular infotainment remains underexplored.

Recent research has explored aerial network support to address these challenges. Drone-mounted base stations offer flexibility and rapid deployment, enhancing network coverage and reliability in vehicular environments [1,9]. Applications include network extension [1], intelligent computation offloading [10], and real-time coordination [11,12]. Complementing these, HAPs such as long-endurance solar drones and stratospheric balloons provide wide-area persistent coverage, surpassing traditional satellite systems in latency and accessibility [13,14,15,16]. While high-speed optical laser communication between aerial platforms delivers exceptional data rates [17,18], atmospheric conditions necessitate hybrid optical–radio systems to maintain link reliability [19]. Despite these advances, existing aerial solutions remain largely isolated from ground vehicular networks and lack optimization for efficient media delivery.

Parallel progress in video transmission technology has yielded adaptive techniques that adjust to varying network conditions. Scalable video coding (SVC) enables prioritized delivery of essential base layers with optional enhancement layers to optimize bandwidth use [20,21,22,23]. Viewport-dependent streaming reduces bandwidth by transmitting only the visible portion of 360° videos [24]. Dynamic bitrate adaptation further improves resource utilization [25,26]. However, these content adaptation strategies generally assume relatively stable networks, limiting their effectiveness in highly dynamic vehicular scenarios.

This review identifies three main limitations in current research: (1) fragmented integration between aerial and terrestrial systems, (2) insufficient coordination between network resource management and content adaptation, and (3) predominantly reactive approaches to congestion management. Vehicle-to-vehicle VLC has demonstrated potential for localized high-speed data exchange [27], yet its integration into heterogeneous aerial–ground networks remain unexplored. As summarized in Table 1, existing works address isolated components but fail to offer a comprehensive solution.

Our proposed architecture overcomes these gaps through three key innovations: unified coordination of multi-altitude aerial platforms, dynamic resource allocation tailored to layered video content, and predictive congestion avoidance mechanisms that proactively maintain service performance.

## 3. Research Methodology and Rationale Adopted in This Work

**Figure 1:**
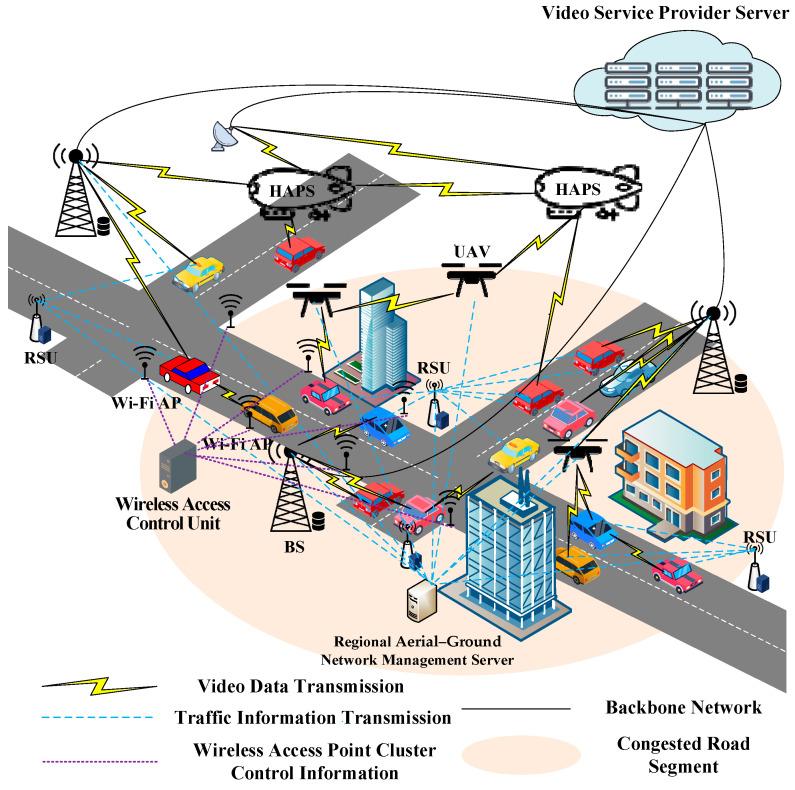
System architecture of aerial–assisted vehicular network for video bandwidth allocation.

The rapid growth of bandwidth-intensive infotainment services in EVs has introduced critical challenges in maintaining user satisfaction within congested urban environments. Our research addresses these challenges through an intelligent system that dynamically adapts video delivery based on real-time factors such as EV location, network congestion, and service priority. Figure 1 illustrates the proposed architecture, which integrates terrestrial and aerial network components to alleviate bandwidth limitations on crowded roadways.

Central to our framework are predictive capabilities that anticipate traffic patterns and connectivity disruptions, enabling proactive strategies such as preloading content before EVs enter areas with poor coverage. The architecture assumes deployment of terrestrial mmWave and THz base stations alongside distributed antenna clusters that deliver video streams directly to EV passengers. RSUs placed along road segments continuously monitor EV movement to support dynamic network adjustments. The network area is partitioned into geographical blocks, each managed by a regional aerial–ground integrated network management server responsible for tracking and coordinating all infrastructure elements—including terrestrial base stations, access points, and aerial nodes—and managing routing and multicast distribution within its domain.

The system leverages both existing and emerging wireless technologies to meet diverse service demands. Ground networks utilize high-frequency mmWave and THz stations complemented by distributed antennas, while aerial support consists of two layers: low-altitude drones (operating below 1 km altitude) providing targeted coverage, and high-altitude platforms (approximately 20 km altitude) offering wide-area persistent connectivity. These aerial nodes function as intelligent relays; high-altitude platforms maintain stable laser links with each other, while hybrid optical–radio links ensure reliable communication with drones and ground stations [15,28]. The system continuously monitors link quality metrics such as received optical signal strength and atmospheric visibility (e.g., fog density). When the FSO link degrades below a defined threshold (e.g., under 60% link margin), the system initiates a seamless handover to RF mode to maintain connectivity. This switching logic is embedded in the aerial relay management module and occurs at sub-second timescales to preserve service continuity. This layered approach enhances connectivity reliability in challenging urban canyon environments where conventional networks often fail.

Video services are organized into three priority tiers, each managed with tailored strategies. Emergency services, such as remote medical consultations, are granted guaranteed bandwidth through a preemptive allocation mechanism similar to priority access in critical applications. Live streaming utilizes adaptive bitrate technology to dynamically adjust video quality based on available network capacity, while non-live entertainment content is delivered on a best-effort basis with intelligent pre-fetching during expected congestion periods. Advanced video encoding techniques separate essential base layers from optional enhancement layers, enabling graceful quality degradation under constrained network resources. For immersive 360° video, viewport-adaptive streaming optimizes bandwidth usage by prioritizing the user’s current field of view.

A key innovation is the dynamic routing system that continuously evaluates multiple delivery paths. When terrestrial networks experience congestion, the management servers redirect traffic through optimal aerial routes via drones or high-altitude platforms. Path selection accounts for real-time factors such as weather conditions affecting laser link quality, node availability, and load balancing needs. Additionally, efficient multicast distribution [29] reduces redundant transmissions when multiple users request the same content.

**Figure 2:**
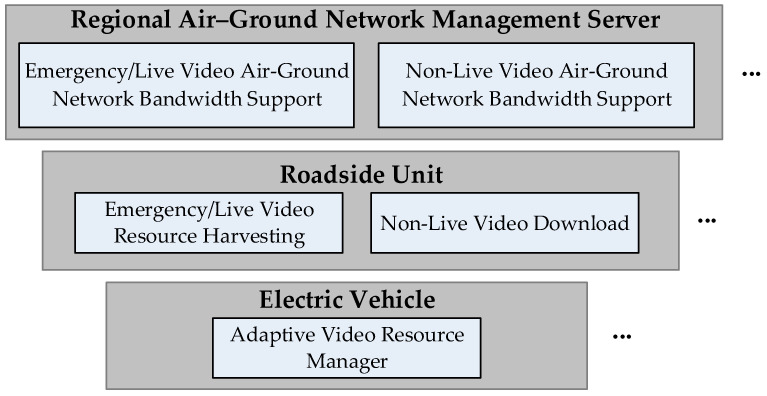
System architecture of aerial network-assisted vehicular network for video bandwidth allocation.

Each EV is equipped with an intelligent communication module that interfaces with the overall network infrastructure. Before starting a trip, users enter their destinations, allowing the system to predict route-specific connectivity challenges. During travel, real-time traffic updates continuously refine these predictions, enabling proactive measures such as strategic content buffering when approaching anticipated coverage gaps. As illustrated at the bottom of Figure 2, upon session initiation, the system assesses both current and projected network conditions along the entire route to determine the optimal delivery method. This selection dynamically prioritizes terrestrial, aerial, or hybrid pathways based on real-time performance metrics.

This comprehensive methodology addresses the limitations of previous works by integrating three critical components: multi-layer coordination of ground and aerial networks, context-aware content adaptation, and predictive resource management. It effectively manages the inherent mobility challenges of vehicular networks, where connection quality varies rapidly across urban environments with diverse infrastructure density. By maintaining multiple redundant delivery options and continuously optimizing resource allocation, the system ensures reliable quality of service even during peak demand in dense urban centers.

The system modules depicted in Figure 2 function as follows.

### 3.1. Adaptive Video Resource Manager Module

**Figure 3:**
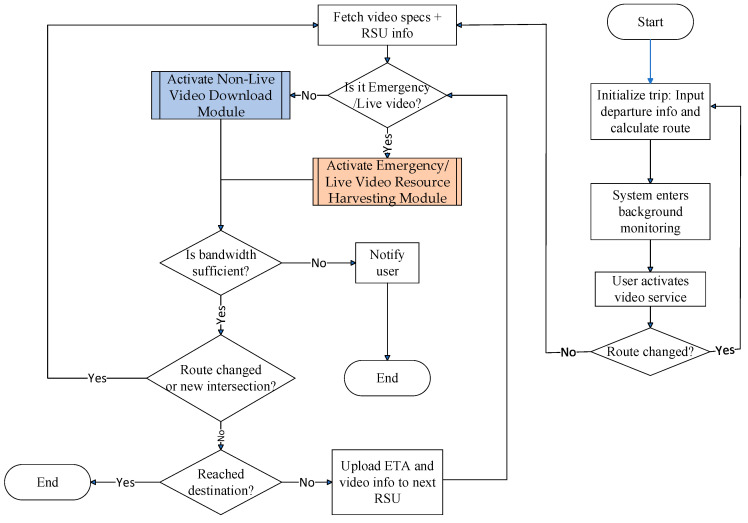
Flowchart of the adaptive video resource manager module.

This module activates during pre-trip configuration, engaging automatically when the passenger inputs departure parameters (location, time, and destination) through the EV’s interface system. Initially, navigation software such as Google Maps determines the EV’s driving route. As illustrated in Figure 3, once a video service is initiated during travel, this module transmits the video application’s specification requirements to the video service provider’s server. It then retrieves the corresponding application specifications, encoding methods, and multicast tree node information from this server. This triggers the EV to upload route-specific multicast node topology and video streaming specifications to roadside infrastructure. Each RSU responds by instantiating the corresponding bandwidth management modules appropriate for the requested video application class.

To address bandwidth fluctuations during peak usage periods, the system adaptively adjusts video resolution and frame rate as needed. However, to ensure the reliability of critical services—such as emergency video transmissions for remote medical procedures—these high-priority applications are given precedence. The system first reduces the quality parameters of lower-priority streams, such as general live or non-live videos, thereby preserving the performance of essential services. This prioritization strategy ensures the fidelity of critical applications while efficiently managing limited bandwidth to support diverse video service demands.

This module flows through the following steps:

Step 1: Prior to initiating the EV’s journey, the system retrieves historical traffic data stored in the EV’s onboard database. Based on the user-defined parameters—including the origin, departure time, and intended destination—the navigation system calculates the optimal driving route.
  (1) $$R^{\sigma }=\left(e_{1}^{\sigma },e_{2}^{\sigma },\cdots ,{e_{i}^{\sigma },\cdots ,e}_{F^{\sigma }-1}^{\sigma },e_{F^{\sigma }}^{\sigma }\right),\;1\leq i\leq F^{\sigma },$$
  (2) $$e_{1}^{\sigma }=O^{\sigma },\;\;\;e_{F^{\sigma }}^{\sigma }=T^{\sigma },$$
  Here, $O^{\sigma }$ and $T^{\sigma }$ denote the origin and destination of EV σ, respectively. $e_{1}^{\sigma }$, $e_{i}^{\sigma }$, and $e_{F^{\sigma }}^{\sigma }$ represent the starting point of the EV’s driving route, the *i*-th waypoint, and terminus of the route, respectively. These two equations define the route structure as an ordered list of segments and are used for logical referencing throughout the system. Since the route is obtained via third-party navigation systems (e.g., Google Maps API), the formulation reflects how the path is internally represented, rather than being derived from first principles.
Step 2: This module transitions into background execution mode, continuously monitoring the system state.
Step 3: If the travel plan changes during transit, the system responds by re-executing Step 1 to generate a revised route incorporating the changes.
Step 4: The system performs an initial congestion evaluation when the passenger activates video streaming, determining if the EV’s route intersects with peak-time traffic bottlenecks. Subsequently, it communicates with the video service provider’s server to retrieve the relevant streaming specifications, including encoding formats and multicast relay node information.
Step 5: The system calculates the intersection that can be reached within ϰ time from the current road segment:
  (3) $$\underset{\bar{\omega }}{arg}\;Max\left({rt}_{c_{\bar{\omega }}^{\sigma }}-{rt}_{c_{1}^{\sigma }}\right),$$
  subject to:
  (4) $$R^{\sigma }=\left(c_{1}^{\sigma },c_{2}^{\sigma },\cdots ,{c_{i}^{\sigma },\ldots ,c}_{\eta^{\sigma }-1}^{\sigma },c_{\eta^{\sigma }}^{\sigma }\right),\;1\leq i\leq \eta^{\sigma },$$
  (5) $$1\leq \bar{\omega }\leq \eta^{\sigma },$$
  (6) $${rt}_{c_{\bar{\omega }}^{\sigma }}-{rt}_{c_{1}^{\sigma }}\leq Min\left[\varkappa ,\left({rt}_{c_{\eta^{\sigma }}^{\sigma }}-{rt}_{c_{1}^{\sigma }}\right)\right]$$
  (7) $$c_{1}^{\sigma }=N^{\sigma },\;\;c_{\eta^{\sigma }}^{\sigma }=T^{\sigma },$$
  (8) $$rt_{c_{1}^{\sigma }}=Cur,$$
  In these expressions, $R^{\sigma }$ defines the EV’s planned route from the current location $N^{\sigma }$ to the destination $T^{\sigma }$. ${rt}_{c_{i}^{\sigma }}$ denotes the estimated arrival time at waypoint $c_{i}^{\sigma }$, and *Cur* is the system timestamp at evaluation time. The optimization in Equation (3) is a heuristic for finding the most forward intersection that can be reached within the available reservation window ϰ. This enables predictive bandwidth allocation at RSUs ahead of the EV’s trajectory. These constraints operationalize the trade-off between lookahead planning and real-time mobility, and are not based on theoretical derivations but on logical control conditions specific to our system architecture.
Step 6: Each RSU along the EV’s route receives estimated arrival time, video specs, and multicast node configurations.
Step 7: If the EV passenger’s application type is emergency/live streaming, the “Emergency/Live Video Resource Harvesting Module” is activated. Otherwise, the “Non-Live Video Download Module” is activated.
Step 8: If the bandwidth is insufficient, the EV passenger is notified that the service cannot be executed and this module is terminated.
Step 9: Should the passenger change the planned route, the system updates its operations by re-executing Step 5 of the process.
Step 10: Prior to arriving at the next intersection, the system verifies whether the upcoming intersection corresponds to the EV’s designated destination.
  (9) $$\bar{\omega }=\bar{\omega }+1,$$
  (10) $$A^{\sigma }=\left\{\begin{array}{cc} 1 & if\;\bar{\omega }=\eta^{\sigma }\\ 0 & otherwise \end{array}\right.,$$
  Here, $\bar{\omega }$ tracks the current position in the waypoint list, and $A^{\sigma }$ is a binary indicator denoting whether the destination has been reached. These logical expressions control the iteration and module continuation. They do not require mathematical derivation but are integral to the heuristic loop structure governing video resource management.
Step 11: If $A^{\sigma }=0$, indicating that the EV has not yet reached its final destination, the system uploads the estimated arrival time at intersection $\bar{\omega }$, together with the associated video application specifications and multicast tree node details, to the RSU overseeing that roadway. The process then returns to Step 7 to resume execution.

### 3.2. Emergency/Live Video Resource Harvesting Module

The Emergency/Live Video Resource Harvesting Module dynamically reallocates bandwidth to high-priority streams, including emergency and live video, when terrestrial resources are insufficient. It operates as follows.

**Figure 4:**
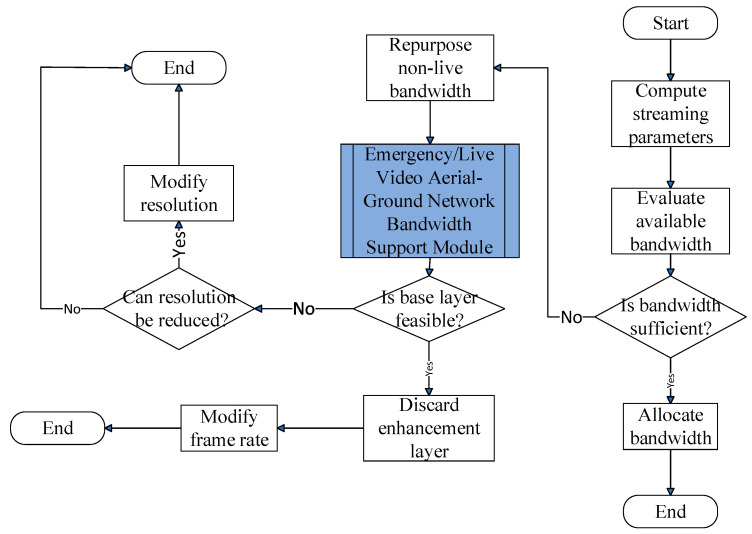
Flowchart of the Emergency/Live Video Resource Harvesting Module.

As illustrated in Figure 4, upon receiving the EV’s video specifications and multicast tree node details, this module first checks whether the reserved bandwidth on the EV’s path meets the requirements for emergency/live service streaming. If insufficient, the system reallocates bandwidth from non-live services to prioritize the emergency/live request.

If bandwidth remains insufficient, the RSU will prioritize multicast tree nodes based on information from the EV and sequentially notify regional aerial–ground network management servers to activate the “Emergency/Live Video Aerial-Ground Network Bandwidth Support Module”. This process will continue until the emergency or live video bandwidth requirements are fulfilled or all available bandwidth has been allocated. Since emergency infotainment services are of paramount importance, if the bandwidth demand for an emergency service still cannot be met, this module will reallocate bandwidth from other live video streams managed by base stations or distributed access point clusters to the emergency application and adjust the bandwidth distribution for the affected streams.

This module flows through the following steps:

Step 1: Using the EV’s computed segment arrival times and infotainment service specifications, the following equations are implemented:
  (11) $${ul}_{t_{j}^{v}}=l^{v},\;1\leq j<E^{v},\;1\leq l^{v},\;if\;\gamma^{v}=1,$$
  (12) $${dl}_{t_{j}^{v}}=l^{v},\;1\leq j<E^{v},\;1\leq l^{v},$$
  (13) $${ur}_{t_{j}^{v}}={vr}_{q^{v}},\;1\leq j<E^{v},\;1\leq q^{v}\leq {\bar{q}}^{v},\;\;if\;\gamma^{v}=1,$$
  (14) $${dr}_{t_{j}^{v}}={vr}_{q^{v}},\;1\leq j<E^{v},\;1\leq q^{v}\leq {\bar{q}}^{v},$$
  (15) $${uf}_{t_{j}^{v}}={vf}_{f^{v}},\;1\leq j<E^{v},\;1\leq f^{v}\leq {\bar{f}}^{v},if\;\gamma^{v}=1,$$
  (16) $${df}_{t_{j}^{v}}={vf}_{f^{v}},\;1\leq j<E^{v},\;1\leq f^{v}\leq {\bar{f}}^{v},$$
  (17) $$t_{j+1}^{v}=t_{j}^{v}+∆,\;1\leq j<E^{v},$$
  (18) $${rt}_{c_{1}^{\sigma }}\leq t_{1}^{v}{\leq t}_{E^{v}}^{v}\leq {rt}_{c_{\eta^{\sigma }}^{\sigma }}.$$
  The binary indicator $\gamma^{v}$ specifies whether the emergency or live video stream v is bidirectional. Specifically, if $\gamma^{v}=1$, the stream is bidirectional; otherwise, if $\gamma^{v}=0$, it is considered unidirectional. The variables $t_{1}^{v}$ and $t_{E^{v}}^{v}$ represent the initial and terminal timestamps of video stream v, respectively. In addition, $c_{i}^{\sigma }$ and $c_{\eta^{\sigma }}^{\sigma }$ denote the *i*-th waypoint index and terminal destination index, respectively, in σ’s planned route. The parameters ${ul}_{t_{j}^{v}}$ and ${df}_{t_{j}^{v}}$ indicate the number of encoding layers used for video upload and download, respectively, at time slot $t_{E^{v}}^{v}$, while ${ur}_{t_{j}^{v}}$ and ${dr}_{t_{j}^{v}}$ denote the video resolution for upload and download. ${uf}_{t_{j}^{v}}$ and ${df}_{t_{j}^{v}}$ track how many frames per second are being uploaded and downloaded for video *v*. $l^{v}$ enumerates the complete set of video layers, including the mandatory base layer and all optional enhancement layers. The resolution is classified into ${\bar{q}}^{v}$ levels, with ${vr}_{q^{v}}$ denoting the resolution level $q^{v}$ specified by the EV passenger for service streaming. Similarly, the video frame rate is classified into ${\bar{f}}^{v}$ levels, with ${vf}_{f^{v}}$ representing the frame rate level $f^{v}$.
  This step defines the EV’s video service requirements over time through Equations (11)–(18), which model how the video stream is structured along the EV’s route. For each trip segment (denoted by time intervals $t_{j}^{v}$), the system determines the number of video layers to upload or download, the resolution level representing video quality, and the frame rate indicating playback smoothness. This enables precise tracking of video service demands throughout the EV’s journey.
Step 2: This step determines time-sliced bandwidth needs using the emergency/live video stream parameters throughout the EV’s trip. It then assesses whether the cumulative bandwidth provisioned by base stations and distributed access point clusters along the planned route is sufficient to satisfy the minimum requirements for uninterrupted video transmission.
  (19) $${dub}_{t_{j}^{v}}=\gamma^{v}\cdot \left[\sum_{\theta }U_{t_{j}^{v}}^{\theta }+\sum_{\hat{\theta }}U_{t_{j}^{v}}^{\hat{\theta }}-\sum_{r=1}^{{ul}_{t_{j}^{v}}}{BS}^{r}\left({uf}_{t_{j}^{v}},{ur}_{t_{j}^{v}}\right)\right],\;1\leq j\leq E^{v},\;{\;rt}_{c_{i}^{\sigma }}\leq t_{j}^{v}\leq {rt}_{c_{i+1}^{\sigma }},$$
  (20) $${ursu}_{t_{j}^{v}}=\left\{\begin{array}{cc} {rsu}_{c_{i}^{\sigma }} & {if\;{dub}_{t_{j}^{v}}<0,\;rt}_{c_{i}^{\sigma }}\leq t_{j}^{v}\leq {rt}_{c_{i+1}^{\sigma }},\;1\leq j\leq E^{v},\;1\leq i<\eta^{\sigma },\\ 0 & otherwise \end{array}\;\right.$$
  (21) $${ddb}_{t_{j}^{v}}=\sum_{\theta }D_{t_{j}^{v}}^{\theta }+\sum_{\hat{\theta }}D_{t_{j}^{v}}^{\hat{\theta }}-\sum_{r=1}^{{dl}_{t_{j}^{v}}}{BS}^{r}\left({df}_{t_{j}^{v}},{dr}_{t_{j}^{v}}\right),\;1\leq j\leq E^{v},\;{\;rt}_{c_{i}^{\sigma }}\leq t_{j}^{v}\leq {rt}_{c_{i+1}^{\sigma }},$$
  (22) $${drsu}_{t_{j}^{v}}=\left\{\begin{array}{cc} {rsu}_{c_{i}^{\sigma }} & {if\;{ddb}_{t_{j}^{v}}<0,\;rt}_{c_{i}^{\sigma }}\leq t_{j}^{v}\leq {rt}_{c_{i+1}^{\sigma }},\;1\leq j\leq E^{v},1\leq i<\eta^{\sigma }\\ 0 & otherwise \end{array},\;\right.$$
  where the indices θ and $\hat{\theta }$ denote the base station and the member of the distributed access point cluster that provide the uplink bandwidth $U_{t_{j}^{v}}^{\theta }$ and $U_{t_{j}^{v}}^{\hat{\theta }}$ for bidirectional video v, respectively. Correspondingly, $D_{t_{j}^{v}}^{\theta }$ and $D_{t_{j}^{v}}^{\hat{\theta }}$ represent the downlink bandwidth allocated by θ and $\hat{\theta }$ to video v. ${BS}^{r}\left(\cdot \right)$ denotes the bandwidth requirement for video encoding, where r corresponds to the index of the base or enhancement layer. The term $c_{i}^{\sigma }$ denotes the index of the RSU overseeing the roadway $c_{i}^{\sigma }$ through which the EV passes.
  Specifically, Equation (19) calculates the uplink bandwidth gap ${dub}_{t_{j}^{v}}$, which compares the total uplink bandwidth allocated by base stations and distributed access point clusters (via $U_{t_{j}^{v}}^{\theta }$ and $U_{t_{j}^{v}}^{\hat{\theta }}$) against the sum of bandwidth requirements for each encoded video layer (up to ${ul}_{t_{j}^{v}}$) based on resolution and frame rate (${{ur}_{t_{j}^{v}},uf}_{t_{j}^{v}}$). When this gap is negative, Equation (20) identifies the RSU (${ursu}_{t_{j}^{v}}$) responsible for the affected road segment $c_{i}^{\sigma }$. Equations (21) and (22) follow a parallel structure for the downlink, defining ${ddb}_{t_{j}^{v}}$ and the corresponding RSU index ${drsu}_{t_{j}^{v}}$ if there is a shortfall in downlink capacity.
  Together, these formulations systematically evaluate where bandwidth is insufficient and which RSU must respond. When a shortfall is detected, the RSU triggers a clustering mechanism [30] to dynamically select an optimal access point set ($\hat{\theta }$). This enables adaptive, distributed bandwidth control to meet the EV’s real-time video demands across each segment.
Step 3: If sufficient bandwidth is available for the requested emergency/live video service, this step allocates the required resources to the EV. Upon successful allocation confirmation, the module terminates its operation.
Step 4: If the bandwidth provided by base stations or distributed access point clusters is inadequate for supporting the required emergency or live service stream, the system investigates whether it is possible to reassign bandwidth originally reserved for non-live services within the EV’s communication range, as outlined in the subsequent procedures.
  (23) $${bub}_{t_{j}^{v}}=\left\{\begin{array}{cc} \sum_{\mu }\left(\sum_{\varphi^{\mu }}U_{t_{j}^{v}}^{\varphi^{\mu }}+\sum_{{\hat{\varphi }}^{\mu }}U_{t_{j}^{v}}^{{\hat{\varphi }}^{\mu }}\right) & if\;{dub}_{t_{j}^{v}}<0\\ 0 & otherwise \end{array}\right.,\;\;1\leq j\leq E^{v},$$
  (24) $${dub}_{t_{j}^{v}}={dub}_{t_{j}^{v}}+{bub}_{t_{j}^{v}}$$
  (25) $${bdb}_{t_{j}^{\sigma ,v}}=\left\{\begin{array}{cc} \sum_{\mu }\left(\sum_{\varphi^{\mu }}D_{t_{j}^{v}}^{\varphi^{\mu }}+\sum_{{\hat{\varphi }}^{\mu }}D_{t_{j}^{v}}^{{\hat{\varphi }}^{\mu }}\right) & if\;{ddb}_{t_{j}^{v}}<0,\\ 0 & otherwise \end{array}\;\right.,\;\;1\leq j\leq E^{v},$$
  (26) $${ddb}_{t_{j}^{v}}={ddb}_{t_{j}^{v}}+{bdb}_{t_{j}^{v}}.$$
  Here, μ represents the index of non-live video. $\varphi^{\mu }$ and ${\hat{\varphi }}^{\mu }$ denote the base station and distributed access point cluster members that have been allocating bandwidth to μ, respectively. $U_{t_{j}^{v}}^{\varphi^{\mu }}$ and $U_{t_{j}^{v}}^{{\hat{\varphi }}^{\mu }}$ represent the uplink bandwidth that $\;\varphi^{\mu }$ and ${\hat{\varphi }}^{\mu }$ can allocate to bidirectional video v when the EV is within their transmission range at time slot $t_{j}^{v}$. Similarly, $D_{t_{j}^{v}}^{\varphi^{\mu }}$ and $D_{t_{j}^{v}}^{{\hat{\varphi }}^{\mu }}$ represent the downlink bandwidth that $\varphi^{\mu }$ and $\hat{\varphi }$ can allocate to video v. ${bub}_{t_{j}^{v}}$ and ${bdb}_{t_{j}^{v}}$ respectively denote the amount of uplink and downlink bandwidth that can be reassigned from non-live video streams to support emergency or live video transmissions.
  Equations (23) and (25) calculate the reassignable uplink (${bub}_{t_{j}^{v}}$) and downlink (${bdb}_{t_{j}^{,v}}$) bandwidth, respectively. These quantities sum the available bandwidth originally allocated to non-live video sessions (indexed by μ) from nearby base stations ($\varphi^{\mu }$) and access point clusters (${\hat{\varphi }}^{\mu }$) at time $t_{j}^{v}$, but only if a bandwidth shortfall (${dub}_{t_{j}^{v}}$ < 0 < 0 or ${ddb}_{t_{j}^{v}}$ < 0) is detected.
  Equations (24) and (26) then update the uplink and downlink shortfall metrics by incorporating the reassigned bandwidth. This process ensures that critical video services (e.g., emergency feeds) can opportunistically borrow bandwidth from non-critical streams without violating system constraints.
  In summary, Step 4 provides a fallback mechanism that temporarily reallocates bandwidth from non-live services to maintain uninterrupted delivery of high-priority live video content.
Step 5: Once bandwidth has been successfully reallocated to meet the minimum requirements for priority stream *v*, the system notifies the originating wireless infrastructure nodes to reassign the bandwidth from *μ* to *v*. It then calls the “Non-Live Video Download Module” to reallocate the necessary bandwidth for the non-live service *μ.* Upon completing the reallocation, the module terminates its execution.
Step 6: If ${dub}_{t_{j}^{v}}$ or ${ddb}_{t_{j}^{v}}$ is negative for a given time slot, the system consults the regional aerial–ground network management server—responsible for managing multicast tree nodes—to determine whether base stations or distributed access point cluster members at each node can supply additional bandwidth.
Step 7: After receiving replies from the regional aerial–ground network management servers, the system sets the priority order for bandwidth allocation from the base stations and distributed access point clusters at the multicast tree nodes to the emergency/live video according to the following formulas:
  (27) $${UMT}^{j,r}=\left[u_{1}^{j,r},u_{2}^{j,r},\cdots ,{u_{k}^{j,r},\cdots ,u}_{T^{v,j}-1}^{j,r},u_{T^{v,j}}^{j,r}\right],\;\;1\leq k<T^{j,r},1\leq j\leq E^{v},1\leq r\leq {ul}_{t_{j}^{v}},\;\;{if\;dub}_{t_{j}^{v}}<0,$$
  (28) $${DMT}^{j,r}=\left[d_{1}^{j,r},d_{2}^{j,r},\cdots ,{d_{\hat{k}}^{j,r},\cdots ,d}_{{\hat{T}}^{v,j}-1}^{j,r},d_{{\hat{T}}^{v,j}}^{j,r}\right],\;1\leq \hat{k}<{\hat{T}}^{j,r},1\leq j\leq E^{v},1\leq r\leq {dl}_{t_{j}^{v}},\;\;{if\;ddb}_{t_{j}^{v}}<0.$$
  These sequences are determined by the following conditions:
  (29) $$\sum_{\psi^{u_{k-1}^{j,r}}}U_{t_{j}^{v}}^{\psi^{u_{k-1}^{j,r}}}+\sum_{{\hat{\psi }}^{u_{k-1}^{j,r}}}U_{t_{j}^{v}}^{{\hat{\psi }}^{u_{k-1}^{j,r}}}\leq \sum_{\psi^{u_{k}^{j,r}}}U_{t_{j}^{v}}^{\psi^{u_{k}^{j,r}}}+\sum_{{\hat{\psi }}^{u_{k}^{j,r}}}U_{t_{j}^{v}}^{{\hat{\psi }}^{u_{k}^{j,r}}},\;1<k\leq T^{j,r},1\leq j\leq E^{v},1\leq r\leq {ul}_{t_{j}^{v}},\;\;{if\;dub}_{t_{j}^{v}}<0,$$
  (30) $$\sum_{\psi^{d_{\hat{k}-1}^{j,r}}}D_{t_{j}^{v}}^{\psi^{d_{\hat{k}-1}^{j,r}}}+\sum_{{\hat{\psi }}^{d_{\hat{k}-1}^{j,r}}}D_{t_{j}^{v}}^{{\hat{\psi }}^{d_{\hat{k}-1}^{j,r}}}\leq \sum_{\psi^{d_{\hat{k}}^{j,r}}}D_{t_{j}^{v}}^{\psi^{d_{\hat{k}}^{j,r}}}+\sum_{{\hat{\psi }}^{d_{\hat{k}}^{j,r}}}D_{t_{j}^{v}}^{{\hat{\psi }}^{d_{\hat{k}}^{j,r}}},\;\;1<\hat{k}\leq {\hat{T}}^{j,r},1\leq j\leq E^{v},1\leq r\leq {dl}_{t_{j}^{v}},\;\;{if\;ddb}_{t_{j}^{v}}<0.$$
  Here, ${UMT}^{j,r}$ and ${DMT}^{j,r}$ represent the sequences of multicast tree nodes that can allocate uplink and downlink bandwidth to video v at time $t_{j}^{v}$, respectively. These sequences are sorted in descending order based on the bandwidth available at each node for the EV at time $t_{j}^{v}$. $T^{j,r}$ and ${\hat{T}}^{j,r}$ denote the number of nodes in the multicast trees ${UMT}^{j,r}$ and ${DMT}^{j,r}$, respectively. $u_{k}^{j,r}$ and $d_{\hat{k}}^{j,r}$ stand for the positions of the $\hat{k}$-th nodes in ${UMT}^{j,r}$ and ${DMT}^{j,r}$, respectively.
The four equations used at this step are designed to prioritize the selection of multicast tree nodes for bandwidth allocation, based on their real-time capacity to support emergency or live video streaming. Equations (27) and (28) define sorted sequences of uplink and downlink nodes, respectively, with the order determined by conditions (29) and (30), which compare cumulative bandwidth availability across candidate nodes. The sorting logic ensures that the most capable nodes—those contributing the highest usable bandwidth—are considered first. This approach embeds a greedy heuristic into the multicast selection process, optimizing throughput and minimizing transmission latency without requiring global optimization. By formulating these priorities explicitly, the framework balances responsiveness with computational efficiency and maintains adaptability across dynamic network states.
Step 8: Based on the priority order of the multicast tree nodes, the system calculates the number of nodes that can provide bandwidth support:
  (31) $${dub}_{t_{j}^{v}}={dub}_{t_{j}^{v}}+\underset{\zeta^{j,r}}{arg}\;Min\left[\sum_{k=1}^{\zeta^{j,r}}\left(\sum_{\psi^{u_{k}^{j,r}}}U_{t_{j}^{v}}^{\psi^{u_{k}^{j,r}}}+\sum_{{\hat{\psi }}^{u_{k}^{j,r}}}U_{t_{j}^{v}}^{{\hat{\psi }}^{u_{k}^{j,r}}}\right),{-dub}_{t_{j}^{v}}\right],\;1\leq k\leq T^{j,r},1\leq j\leq E^{v},1\leq r\leq {ul}_{t_{j}^{v}},\;\;{if\;dub}_{t_{j}^{v}}<0,$$
  (32) $$\begin{array}{cc} {ddb}_{t_{j}^{v}}={ddb}_{t_{j}^{v}}+ & \underset{{\hat{\zeta }}^{j,r}}{arg}\;Min\left[\sum_{\hat{k}=1}^{{\hat{\zeta }}^{j,r}}\left(\sum_{\psi^{d_{\hat{k}}^{j,r}}}D_{t_{j}^{v}}^{\psi^{d_{\hat{k}}^{j,r}}}+\sum_{{\hat{\psi }}^{d_{\hat{k}}^{j,r}}}D_{t_{j}^{v}}^{{\hat{\psi }}^{d_{\hat{k}}^{j,r}}}\right),{-ddb}_{t_{j}^{v}}\right],\\ & 1\leq \hat{k}\leq {\hat{T}}^{j,r},1\leq j\leq E^{v},{1\leq r\leq {dl}_{t_{j}^{v}},\;\;if\;ddb}_{t_{j}^{v}}<0. \end{array}$$
  Here, $\zeta^{j,r}$ and ${\hat{\zeta }}^{j,r}$ represent the number of multicast tree nodes supporting uplink and downlink bandwidth, respectively. $\psi^{u_{k}^{j,r}}$ and ${\hat{\psi }}^{u_{k}^{j,r}}$ stand for the base stations and distributed access point cluster members covering the node $u_{k}^{j,r}$ in the sequence ${UMT}^{j,r}$. $U_{t_{j}^{v}}^{\psi^{u_{k}^{j,r}}}$ and $U_{t_{j}^{v}}^{{\hat{\psi }}^{u_{k}^{j,r}}}$ denote the uplink bandwidth that $\psi^{u_{k}^{j,r}}$ and ${\hat{\psi }}^{u_{k}^{j,r}}$ can allocate to bidirectional video v at time slot $t_{j}^{v}$. Similarly, $\psi^{d_{\hat{k}}^{j,r}}$ and ${\hat{\psi }}^{d_{\hat{k}}^{j,r}}$ represent the base stations and distributed access point cluster members covering node $d_{\hat{k}}^{j,r}$ in sequence ${DMT}^{j,r}$, while $D_{t_{j}^{v}}^{\psi^{d_{\hat{k}}^{j,r}}}$ and $D_{t_{j}^{v}}^{{\hat{\psi }}^{d_{\hat{k}}^{j,r}}}$ denote the downlink bandwidth that $\psi^{d_{\hat{k}}^{j,r}}$ and ${\hat{\psi }}^{u_{k}^{j,r}}$ can allocate to video v at time slot $t_{j}^{v}$.
  Equations (31) and (32) compute the total bandwidth that can be leveraged by selectively including an appropriate number of high-priority nodes from the multicast tree sequences identified in the previous step. By incrementally summing the bandwidth from top-ranked nodes in ${UMT}^{j,r}$ and ${DMT}^{j,r}$, the equations determine the minimum number of contributors ($\zeta^{j,r}$ and ${\hat{\zeta }}^{j,r}$) required to satisfy the uplink and downlink demands of video *v*. The arg Min[·] operation ensures a balance between efficiency and sufficiency by terminating the accumulation process as soon as the aggregated capacity reaches or slightly exceeds the outstanding bandwidth deficit. This approach minimizes resource consumption while ensuring that video transmission requirements are satisfied. Overall, the formulation implements a localized optimization mechanism that is both computationally efficient and adaptive to real-time network conditions, consistent with the guiding principles of the proposed heuristic framework.
Step 9: The system sends a request to the regional aerial–ground network management server overseeing the multicast tree nodes identified in the previous step, triggering the “Emergency/Live Video Aerial-Ground Network Bandwidth Support Module”. The server then evaluates whether base stations or distributed access point cluster members under its jurisdiction can relay bandwidth to video v via drones or aerial platforms.
Step 10: If the data relay process successfully meets the requirements of v, this module advances to Step 22 to resume its operation.
Step 11: Remove the first $\zeta^{j,r}$ and ${\hat{\zeta }}^{j,r}$ nodes from ${UMT}^{j,r}$ and ${DMT}^{j,r}$, respectively, and update the ${UMT}^{j,r}$ and ${DMT}^{j,r}$ nodes as follows:
  (33) $$u_{k}^{j,r}=u_{k+l}^{j,r},\;1\leq k\leq T^{j,r}-\zeta^{j,r},1\leq j\leq E^{v},1\leq r\leq {ul}_{t_{j}^{v}},\;{if\;dub}_{t_{j}^{v}}<0,$$
  (34) $$d_{\hat{k}}^{j,r}=d_{\hat{k}+l}^{j,r},\;1\leq \hat{k}\leq {\hat{T}}^{j,r}-{\hat{\zeta }}^{j,r},1\leq j\leq E^{v},\;{1\leq r\leq {dl}_{t_{j}^{v}},if\;ddb}_{t_{j}^{v}}<0,$$
  (35) $$T^{j,r}=T^{j,r}-\zeta^{j,r},\;1\leq j\leq E^{v},{1\leq r\leq {ul}_{t_{j}^{v}},\;if\;dub}_{t_{j}^{v}}<0,$$
  (36) $${\hat{T}}^{j,r}={\hat{T}}^{j,r}-{\hat{\zeta }}^{j,r},\;1\leq j\leq E^{v},1\leq r\leq {dl}_{t_{j}^{v}},{if\;ddb}_{t_{j}^{v}}<0.$$
  As shown in Equations (33)–(36), the head elements of the ${UMT}^{j,r}$ and ${DMT}^{j,r}$ sequences—those already selected to fulfill the bandwidth demand—are removed, and the remaining nodes are reindexed to maintain the structural integrity of the sequences. The values of $T^{j,r}$ and ${\hat{T}}^{j,r}$, which track the total number of candidate nodes, are correspondingly decremented. This update ensures that future iterations of the allocation logic operate only on unassigned resources, thereby preventing redundancy and enforcing efficient use of the remaining network capacity. This iterative pruning aligns with the overall heuristic framework by ensuring adaptability and local optimization in a dynamic bandwidth allocation environment.
Step 12: If the sequences ${UMT}^{j,r}$ and ${DMT}^{j,r}$ have not yet been fully processed (i.e., not completely emptied), the system returns to Step 8 and resumes execution.
Step 13: If video stream v does not contain enhancement layer encoding, the system skips directly to Step 20 for continued processing. Otherwise, it proceeds to the subsequent step.
Step 14: The base layer bandwidth requirement for video stream *v* is calculated as follows:
  (37) $${dub}_{t_{j}^{v}}=\gamma^{v}\cdot \left[{dub}_{t_{j}^{v}}+\sum_{r=2}^{{ul}_{t_{j}^{v}}}{BS}^{r}\left({uf}_{t_{j}^{v}},{ur}_{t_{j}^{v}}\right)\right],\;1\leq j\leq E^{v},\;{\;rt}_{c_{i}^{\sigma }}\leq t_{j}^{v}\leq {rt}_{c_{i+1}^{\sigma }},{if\;dub}_{t_{j}^{v}}<0,$$
  (38) $${ddb}_{t_{j}^{v}}={ddb}_{t_{j}^{v}}+\sum_{r=2}^{{dl}_{t_{j}^{v}}}{BS}^{r}\left({df}_{t_{j}^{v}},{dr}_{t_{j}^{v}}\right),\;1\leq j\leq E^{v},\;{\;rt}_{c_{i}^{\sigma }}\leq t_{j}^{v}\leq {rt}_{c_{i+1}^{\sigma }},{if\;ddb}_{t_{j}^{v}}<0,$$
  Equations (37) and (38) refine the estimated bandwidth requirements for the base layer of video stream *v* during time slot $t_{j}^{v}$. The expressions integrate both initial bandwidth deficits—represented by ${dub}_{t_{j}^{v}}$ and ${ddb}_{t_{j}^{v}}$—and cumulative contributions from additional base stations indexed by *r* = 2 to ${ul}_{t_{j}^{v}}$ or ${dl}_{t_{j}^{v}}$, respectively. The term ${BS}^{r}$(⋅) denotes the *r*-th base station’s bandwidth support within the current uplink or downlink cluster. The scaling factor $\gamma^{v}$ adjusts the effective uplink requirement to account for video-specific transmission demands (e.g., encoding complexity or priority weight). By integrating this hierarchical accumulation mechanism, these formulas ensure that the base layer—a critical component for maintaining minimal video quality—is reliably sustained. This approach aligns with the heuristic’s design by prioritizing robustness and continuity in emergency video delivery under constrained network conditions.
Step 15: If both ${dub}_{t_{j}^{v}}$ and ${ddb}_{t_{j}^{v}}$ are non-negative, it indicates that the bandwidth requirements for the base layer encoding of video stream v have been met. In this case, the system discards the enhancement layer encoding and returns to Step 2, as specified by the following two equations; otherwise, it proceeds to the next step.
  (39) $${ul}_{t_{j}^{v}}=1,\;1\leq j<E^{v},\;\;if\;\gamma^{v}=1,$$
  (40) $${dl}_{t_{j}^{v}}=1,\;1\leq j<E^{v}.$$
  This step serves as a validation checkpoint within the bandwidth allocation process. By confirming that both uplink and downlink base layer bandwidth deficits, ${dub}_{t_{j}^{v}}$ and ${ddb}_{t_{j}^{v}}$, are non-negative, the system ensures that the essential quality of the video stream *v* can be maintained without requiring additional enhancement layers. Setting ${ul}_{t_{j}^{v}}=1$ and ${dl}_{t_{j}^{v}}=1$ effectively signals the system to prioritize base layer transmission exclusively, simplifying resource allocation and reducing network load. This conditional check supports a fail-safe mechanism within the heuristic framework, allowing early termination of more complex allocation steps when base layer needs are already satisfied, thereby improving computational efficiency and responsiveness.
Step 16: If video stream v is identified as a live stream, the system proceeds to Step 20 for further execution. Otherwise, it continues to the subsequent step.
Step 17: Bandwidth that was originally assigned to other live video streams from ground base stations or distributed access point cluster members within the EV’s coverage area to the urgent application is reallocated, as described below:
  (41) $${dub}_{t_{j}^{v}}={dub}_{t_{j}^{v}}+Min\left[\sum_{\mu }\left(\sum_{\varphi^{\mu }}U_{t_{j}^{v}}^{\varphi^{\mu }}+\sum_{{\hat{\varphi }}^{\mu }}U_{t_{j}^{v}}^{{\hat{\varphi }}^{\mu }}\right),{-dub}_{t_{j}^{v}}\right],\;{if\;dub}_{t_{j}^{v}}<0,$$
  (42) $${ddb}_{t_{j}^{v}}={ddb}_{t_{j}^{v}}+Min\left[\sum_{\mu }\left(\sum_{\varphi^{\mu }}D_{t_{j}^{v}}^{\varphi^{\mu }}+\sum_{{\hat{\varphi }}^{\mu }}D_{t_{j}^{v}}^{{\hat{\varphi }}^{\mu }}\right),{-ddb}_{t_{j}^{v}}\right],\;{if\;ddb}_{t_{j}^{v}}<0,$$
  Here, μ represents the index of the live video stream from which bandwidth is being reallocated. $\varphi^{\mu }$ and ${\hat{\varphi }}^{\mu }$ denote the base stations and distributed access point cluster members originally allocating bandwidth to μ. $U_{t_{j}^{v}}^{\varphi^{\mu }}$ and $U_{t_{j}^{v}}^{{\hat{\varphi }}^{\mu }}$ denote the uplink bandwidth that $\varphi^{\mu }$ and $\hat{\varphi }$ can allocate to the urgent video stream v when the EV is within their coverage at time slot $t_{j}^{v}$. Similarly, $D_{t_{j}^{v}}^{\varphi^{\mu }}$ and $D_{t_{j}^{v}}^{{\hat{\varphi }}^{\mu }}$ stand for the downlink bandwidth that $\varphi^{\mu }$ and φ can allocate to the urgent video stream v.
  This step details the process of dynamically reallocating bandwidth from existing live video streams to the emergency video stream *v* within the EV’s coverage area. Equations (41) and (42) calculate the additional uplink and downlink bandwidth that can be reclaimed by summing the resources allocated by base stations and distributed access point clusters currently serving other live streams (indexed by *μ*). The use of the Min[∙] function ensures that the reallocation does not exceed the bandwidth deficit, preventing over-allocation and preserving system stability. This step enables a responsive adjustment of network resources, prioritizing urgent traffic by temporarily borrowing capacity from less critical live streams, thus maintaining efficient utilization of limited bandwidth under dynamic network conditions.
Step 18: If reallocating bandwidth from other live video streams to the emergency service results in the inability to smoothly download and play the affected live service streams, ${dub}_{t_{j}^{v}}$ and ${ddb}_{t_{j}^{v}}$ are set to the bandwidth quota of the affected live service stream and the system returns to Step 6 to request aerial–ground network support for restoring bandwidth to the affected live service stream.
Step 19: Once the bandwidth requirements for the EV passenger’s emergency service are met, the system proceeds to Step 22 to continue execution. Otherwise, it advances to the next step.
Step 20: If the service in use permits frame rate adjustment and the current frame rate has not yet reached the minimum threshold defined by the system, the frame rate is recalibrated according to the following equations. The system then returns to Step 2 to resume execution.
  (43) $${uf}_{t_{j}^{v}}=\left\{\begin{array}{cc} {vf}_{f^{v}-1} & {if\;{ursu}_{t_{j}^{v}}>0,\;f^{v}>1,\;rt}_{c_{i}^{\sigma }}\leq t_{j}^{v}\leq {rt}_{c_{i+1}^{\sigma }},\;1\leq j\leq E^{v},1\leq i<\eta^{\sigma }\\ {vf}_{1} & otherwise \end{array},\right.$$
  (44) $${df}_{t_{j}^{v}}=\left\{\begin{array}{cc} {vf}_{f^{v}-1} & {if\;{drsu}_{t_{j}^{v}}>0,\;f^{v}>1,\;rt}_{c_{i}^{\sigma }}\leq t_{j}^{v}\leq {rt}_{c_{i+1}^{\sigma }},\;1\leq j\leq E^{v},1\leq i<\eta^{\sigma }\\ {vf}_{1} & otherwise \end{array}\right.,$$
  (45) $$f^{v}=f^{v}-1,\;\;{if\;{ursu}_{t_{j}^{v}}>0,\;f^{v}>1,\;rt}_{c_{i}^{\sigma }}\leq t_{j}^{v}\leq {rt}_{c_{i+1}^{\sigma }},\;1\leq j\leq E^{v},1\leq i<\eta^{\sigma }.$$
  Here, ${uf}_{t_{j}^{v}}$ and ${df}_{t_{j}^{v}}$ represent the uplink and downlink frame rates of video v for the EV user. ${ursu}_{t_{j}^{v}}$ and ${drsu}_{t_{j}^{v}}$ indicate the RSU managing the segment between $c_{i}^{\sigma }$ and $c_{i+1}^{\sigma }$ where uplink and downlink bandwidth are insufficient at time slot $t_{j}^{v}$. The variables $t_{1}^{v}$ and $t_{E^{,v}}^{v}$ denote the initial and terminal timestamps of the video, respectively. The term ${vf}_{f^{v}}$ stands for the frame rate level $f^{v}$ specified by the EV passenger for the corresponding service stream.
  This step introduces a dynamic frame rate adjustment mechanism to cope with bandwidth shortages when reallocating resources is insufficient. The equations (43)–(45) define how uplink and downlink frame rates (${uf}_{t_{j}^{v}}$ and ${df}_{t_{j}^{v}}$) are reduced stepwise, guided by the RSU bandwidth insufficiency indicators (${ursu}_{t_{j}^{v}}$ and ${drsu}_{t_{j}^{v}}$). By decrementing the frame rate level $f^{v}$ when necessary—while respecting predefined minimum thresholds—this adaptive approach balances video quality degradation against network capacity constraints. This ensures continuous service availability for the EV passenger’s emergency video stream, preventing abrupt interruptions. Returning to Step 2 after adjustment enables the system to iteratively refine bandwidth allocation and video encoding parameters, thereby maintaining an optimal trade-off between performance and resource limitations under dynamic network conditions.
Step 21: If the service supports resolution adjustment and the current resolution level remains above the system-defined minimum threshold, the system recalibrates the resolution using the following equations and returns to Step 2 to resume execution.
  (46) $${ur}_{t_{j}^{v}}=\left\{\begin{array}{cc} {vr}_{q^{v}-1} & {if\;{ursu}_{t_{j}^{v}}>0,\;q^{v}>1,\;rt}_{c_{i}^{\sigma }}\leq t_{j}^{v}\leq {rt}_{c_{i+1}^{\sigma }},\;1\leq j\leq E^{v},1\leq i<\eta^{\sigma }\\ {vr}_{1} & otherwise \end{array}\right.,$$
  (47) $${dr}_{t_{j}^{v}}=\left\{\begin{array}{cc} {vr}_{q^{v}-1} & {if\;{drsu}_{t_{j}^{v}}>0,\;q^{v}>1,\;rt}_{c_{i}^{\sigma }}\leq t_{j}^{v}\leq {rt}_{c_{i+1}^{\sigma }},\;1\leq j\leq E^{v},1\leq i<\eta^{\sigma }\\ {vr}_{1} & otherwise \end{array}\right.,$$
  (48) $$q^{v}=q^{v}-1,\;\;{if\;{ursu}_{t_{j}^{v}}>0,\;q^{v}>{\underline{q}}^{v},\;rt}_{c_{i}^{\sigma }}\leq t_{j}^{v}\leq {rt}_{c_{i+1}^{\sigma }},\;1\leq j\leq E^{v},1\leq i<\eta^{\sigma }.$$
  In this context, ${ur}_{t_{j}^{v}}$ and ${dr}_{t_{j}^{v}}$ denote the resolution settings for uplink and downlink transmission, respectively, whereas ${vr}_{q^{v}}$ indicates the resolution level $q^{v}$ as requested by the EV passenger for the service stream.
  This step implements an adaptive resolution adjustment mechanism to further optimize bandwidth usage when necessary. Equations (46) and (47) update the uplink and downlink resolution levels (${ur}_{t_{j}^{v}}$ and ${dr}_{t_{j}^{v}}$) by decrementing the current resolution $q^{v}$ when the bandwidth insufficiency flags (${ursu}_{t_{j}^{v}}$ and ${drsu}_{t_{j}^{v}}$) indicate resource constraints and the resolution is above the system-defined minimum. Equation (48) manages the resolution index update to ensure gradual stepping down through predefined resolution levels, avoiding abrupt quality drops. This approach allows the system to maintain video service continuity by trading off resolution quality dynamically, aligned with network conditions and user preferences.
Step 22: If the EV passenger’s service bandwidth requirements have been met, the base stations, distributed access point clusters, or multicast tree nodes involved in the bandwidth allocation process are notified to assign the bandwidth to the EV passenger.
Step 23: The bandwidth allocation results are transmitted back to the EV, and the execution of this module is terminated.

### 3.3. Non-Live Video Download Module

**Figure 5:**
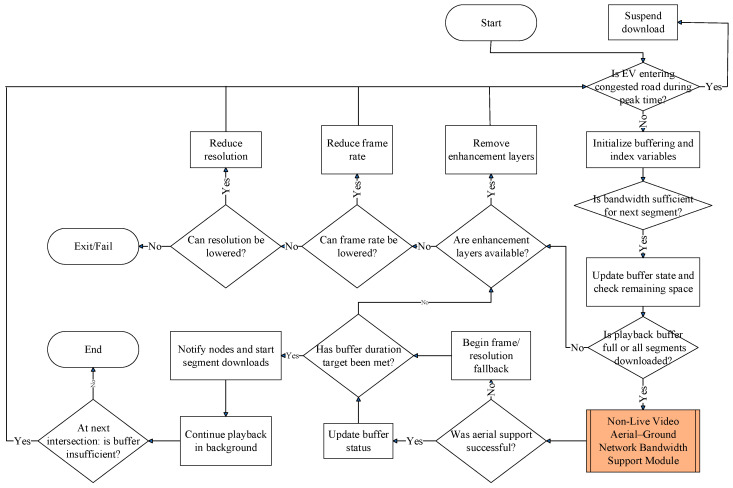
Flowchart of the Non-Live Video Download Module.

As illustrated in Figure 5, upon receiving the EV’s location, non-live video playback requirements, and multicast tree node information, this module initially verifies whether the EV is traversing congested road segments during peak traffic periods. If the EV is not traveling on a congested roadway during peak hours, this module examines whether the base stations or distributed access point clusters surrounding the EV’s route can provide sufficient bandwidth for non-live video users. Because segments of non-live video content are either pre-stored on the service provider’s server or cached at the edge nodes of the multicast tree, the EV is able to proactively download upcoming segments via base stations or distributed access point clusters along its current travel path during playback. This module continuously monitors the remaining buffered video content on the EV’s onboard storage and dynamically adjusts bandwidth allocation from nearby network nodes accordingly. When available bandwidth is insufficient to support video segment downloads, the system activates the “Non-Live Video Aerial-Ground Network Bandwidth Support Module” to identify and utilize suitable multicast nodes for the download process. Additionally, since this study prioritizes bandwidth allocation for emergency/live infotainment services, whenever the EV is traveling through a congested road segment, all non-live video downloads are handled exclusively by the “Non-Live Video Aerial-Ground Network Bandwidth Support Module”.

This module flows through the following steps:

Step 1: If the EV is traveling through a congested road segment during peak hours, the user’s non-live video download request is paused, and the operation of this module is halted.
Step 2: The following variables are initialized:
  (49) $${lp}^{v}=0,$$
  (50) $${SL}^{v}={\bar{SL}}^{v},$$
  (51) $${cl}^{v}=l^{v},\;\;1\leq l^{v},$$
  (52) $${cr}^{v}={vr}_{q^{v}},\;\;1\leq q^{v}\leq {\bar{q}}^{v},$$
  (53) $${cf}^{v}={vf}_{f^{v}},\;\;1\leq f^{v}\leq {\bar{f}}^{v},$$
  where ${lp}^{v}$ represents the index of the last unplayed video segment stored in the EV for video v. ${SL}^{v}$ denotes the remaining playable video duration that can be pre-downloaded by the EV, while ${\bar{SL}}^{v}$ is the system-defined upper limit for pre-downloaded video playback time. The video stream consists of a base layer and one or more enhancement layers. $l^{v}$ is the total number of layers; ${cl}^{v}$, ${cr}^{v}$, and ${cf}^{v}$, respectively, stand for the number of encoding layers, resolution, and frame rate of the video segments. The resolution of *v* is categorized into ${\bar{q}}^{v}$ levels, while the frame rate is categorized into $f^{v}$ levels. ${vr}_{q^{v}}$ and ${vf}_{f^{v}}$ stand for the resolution corresponding to level $q^{v}$ and the frame rate corresponding to level $f^{v}$, respectively.
  This step establishes the initial state variables critical for managing non-live video downloads in dynamic vehicular environments. The variable ${lp}^{v}$ tracks the index of the last unplayed video segment stored onboard, enabling the system to monitor playback progress and buffer status. ${SL}^{v}$ represents the remaining duration of video content that the EV can pre-download, bounded by the system-defined upper limit ${\bar{SL}}^{v}$, which prevents excessive buffering and optimizes storage use. The video encoding parameters—number of layers ${cl}^{v}$, resolution c${cr}^{v}$, and frame rate ${cf}^{v}$—are initialized to reflect the current streaming quality settings. These parameters account for layered video, allowing adaptive adjustment to network conditions. The resolution ${vr}_{q^{v}}$ and frame rate ${vf}_{f^{v}}$ are discretized into levels to facilitate systematic quality control. Collectively, these definitions provide a foundation for dynamically regulating bandwidth allocation and download strategies tailored to the EV’s route, storage, and video playback status, thereby ensuring seamless video delivery despite variable network conditions.
Step 3: If the EV is navigating a congested road segment during peak hours, the system halts the passenger’s non-live video download request and suspends bandwidth allocation. Otherwise, based on the passenger’s non-live video playback settings and the remaining duration of preloaded video segments, this step evaluates whether the available bandwidth is sufficient to preload upcoming video segments using the following formula:
  (54) $$\underset{{ps}^{v}}{arg}\;Max\left|\sum_{\vartheta }D_{\tau^{{lp}^{v}+1}+\delta }^{\vartheta }+\sum_{\hat{\vartheta }}D_{\tau^{{lp}^{v}+1}+\delta }^{\hat{\vartheta }}-\sum_{l={lp}^{v}+1}^{{{lp}^{v}+ps}^{v}}\sum_{r=1}^{{cl}^{v}}{SS}^{r}\left({cf}^{v},{{cr}^{v},cd}_{l}\right)\right|,$$
  The above equation is subject to the following conditions:
  (55) $$\sum_{\vartheta }D_{\tau^{{lp}^{v}+1}+\delta }^{\vartheta }+\sum_{\hat{\vartheta }}D_{\tau^{{lp}^{v}+1}+\delta }^{\hat{\vartheta }}\geq \sum_{l={lp}^{v}+1}^{{{lp}^{v}+ps}^{v}}\sum_{r=1}^{{cl}^{v}}{SS}^{r}\left({cf}^{v},{{cr}^{v},cd}_{l}\right),$$
  (56) $$\sum_{l={lp}^{v}+1}^{{{lp}^{v}+ps}^{v}}{cd}_{l}\leq {SL}^{v},\;\;{{lp}^{v}+ps}^{v}\leq {SC}^{v},$$
  (57) $$c{pt}_{1}^{v}\leq \tau^{l}<\tau^{l}+\delta \leq {cpt}_{l}^{v}<c{pt}_{{SC}^{v}}^{v},\;\;{lp}^{v}+1\leq l\leq {{lp}^{v}+ps}^{v},$$
  (58) $${cpt}_{l-1}^{v}\leq {cpt}_{l}^{v},\;\;\;\;{lp}^{v}+1\leq l\leq {{lp}^{v}+ps}^{v},$$
  (59) $${buf}^{\sigma }+\sum_{l={lp}^{v}+1}^{{{lp}^{v}+ps}^{v}}\sum_{r=1}^{{cl}^{v}}{SS}^{r}\left({cf}^{v},{{cr}^{v},cd}_{l}\right)\leq {\bar{buf}}^{\sigma },$$
  where $\tau^{l}$ is the time at which video segment l is downloaded. $D_{\tau^{l}}^{\vartheta }$ and $D_{\tau^{l}}^{\hat{\vartheta }}$ represent the bandwidth allocated to non-live video segment l from base station ϑ and distributed access point cluster member $\tau^{l}$ at time $\tau^{l}$, respectively. The variable δ denotes the number of consecutive time slots following $\tau^{l}$ that are allocated for the continuous downloading of video segments. ${SC}^{v}$ stands for the total number of video segments in video v, and ${ps}^{v}$ denotes the number of video segments that are preloaded. The variable ${cpt}_{l}^{v}$ represents the duration of the *l*-th video portion, while ${SS}^{r}\left(\cdot \right)$ indicates the bit size of the base or enhancement layer r for a given video portion. The term ${cd}_{l}$ corresponds to the playback duration of segment l. Additionally, ${buf}^{\sigma }$ refers to the remaining buffer capacity available on the EV prior to preloading, and ${\bar{buf}}^{\sigma }$ denotes the total buffer storage capacity of the EV.
  This step evaluates whether the system can continue preloading upcoming non-live video segments for the EV passenger under current network and buffer conditions, or if downloads must be suspended due to congestion. Equation (54) maximizes the preload segment count ${ps}^{v}$ by comparing the available downlink bandwidth aggregated from base stations ($D_{\tau^{l}}^{\vartheta }$) and distributed access points ($D_{\tau^{l}}^{\hat{\vartheta }}$) over a window of consecutive time slots δ against the cumulative video segment size ${SS}^{r}\left(\cdot \right)$ needed for smooth playback. Constraints (55)–(59) ensure that the total allocated bandwidth meets or exceeds the preload demand; the preloaded content does not surpass the remaining buffer space or total video length; segment download times are ordered sequentially within valid playback timeframes; and buffer capacity limits are respected to prevent overflow. Together, this formulation ensures that video preloading is adaptively managed to balance seamless playback and network resource availability, preventing unnecessary bandwidth allocation during congestion or limited buffer scenarios.
Step 4: The following temporary variables are updated:
  (60) $${TSL}^{v}={SL}^{v}-\sum_{l={lp}^{v}+1}^{{{lp}^{v}+ps}^{v}}{cd}_{l},$$
  (61) $${{tbuf}^{\sigma }=buf}^{\sigma }+\sum_{l={lp}^{v}+1}^{{{lp}^{v}+ps}^{v}}\sum_{r=1}^{{cl}^{v}}{SS}^{r}\left({cf}^{v},{{cr}^{v},cd}_{l}\right),$$
  (62) $${tlp}^{v}={{lp}^{v}+ps}^{v}.$$
  Here, ${TSL}^{v}$ represents the updated remaining playable duration that can be pre-downloaded, ${tbuf}^{\sigma }$ denotes the updated remaining buffer storage on the EV, and ${tlp}^{v}$ stands for the index of the last segment remaining in the playback buffer after bandwidth allocation in the prior step.
  This step updates key temporary variables to reflect the impact of the preloading decisions made in Step 3. Equation (60) recalculates ${TSL}^{v}$, the remaining playable video duration that still needs to be downloaded after accounting for the newly preloaded segments. Equation (61) updates the temporary buffer occupancy ${tbuf}^{\sigma }$ by adding the bit sizes of the newly downloaded video segments to the existing buffer level, ensuring accurate tracking of buffer usage. Finally, Equation (62) sets ${tlp}^{v}$ as the index of the latest segment buffered, marking the current boundary between downloaded and yet-to-be-downloaded content. Together, these updates maintain synchronization between buffer status, playback progression, and network resource allocation, supporting adaptive video streaming management in dynamic conditions.
Step 5: If all non-live video portions have been successfully downloaded, or if ${TSL}^{v}$ is greater than or equal to the system-defined minimum playback duration required for preloading, the system proceeds to Step 15 for continued execution. Otherwise, the system advances to the following step.
Step 6: The number of video portions ${\tilde{ps}}^{v}$ that can continue to be downloaded is determined using the following formula:
  (63) $$\underset{{\tilde{ps}}^{v}}{arg}\;Min\left[{\bar{SL}}^{v}-\left(\sum_{l=t{lp}^{v}+1}^{{t{lp}^{v}+\tilde{ps}}^{v}}{cd}_{l}+{TSL}^{v}\right)\right],\;\;\sum_{l=t{lp}^{v}+1}^{{t{lp}^{v}+\tilde{ps}}^{v}}{cd}_{l}+{TSL}^{v}\leq {\bar{SL}}^{v}.$$
  Equation (63) formulates a constrained optimization problem that minimizes the difference between the upper threshold ${\bar{SL}}^{v}$ and the sum of the playback durations of candidate segments plus the current buffered duration ${TSL}^{v}$. This formulation ensures that additional buffering remains within safe limits, preventing resource waste and buffer overflow while still extending playback continuity. It reflects a practical trade-off between aggressive preloading and system stability, especially under fluctuating network conditions.
Step 7: Based on the video multicast tree node information provided by the video service provider, the system consults the regional aerial–ground network management servers responsible for managing the multicast tree nodes. After receiving responses, the system determines the order in which base stations and distributed access point clusters at the multicast tree nodes allocate bandwidth for video portion downloads using the following sequences:
  (64) $${NMT}^{r}=\left[{\tilde{d}}_{1}^{r},{\tilde{d}}_{2}^{r},\cdots ,{{\tilde{d}}_{\tilde{i}}^{r},\cdots ,\tilde{d}}_{{\tilde{T}}^{r}-1}^{r},{\tilde{d}}_{{\tilde{T}}^{r}}^{r}\right],\;1\leq \tilde{i}\leq {\tilde{T}}^{r},1\leq r\leq {cl}_{s^{v}},$$
  (65) $${SMT}^{r}=\left[{\tilde{s}}_{1}^{r},{\tilde{s}}_{2}^{r},\cdots ,{{\tilde{s}}_{\tilde{i}}^{r},\cdots ,\tilde{s}}_{{\tilde{T}}^{r}-1}^{r},{\tilde{s}}_{{\tilde{T}}^{r}}^{r}\right],\;1\leq \tilde{i}\leq {\tilde{T}}^{r},\;1\leq r\leq {cl}_{s^{v}},$$
  (66) $${CMT}^{r}=\left[{\tilde{n}}_{1}^{r},{\tilde{n}}_{2}^{r},\cdots ,{{\tilde{n}}_{\tilde{i}}^{r},\cdots ,\tilde{n}}_{{\tilde{T}}^{r}-1}^{r},{\tilde{n}}_{{\tilde{T}}^{r}}^{r}\right],\;1\leq \tilde{i}\leq {\tilde{T}}^{r},1\leq r\leq {cl}_{s^{v}},$$
  (67) $$\sum_{{\tilde{\delta }}^{{\tilde{d}}_{\tilde{i}}^{r}}}\left(\left(\sum_{\psi^{{\tilde{d}}_{\tilde{i}}^{r}}}{\tilde{D}}_{\tau^{{\tilde{s}}_{\tilde{i}}^{r}}+{\tilde{\delta }}^{{\tilde{d}}_{\tilde{i}}^{r}}}^{\psi^{{\tilde{d}}_{\tilde{i}}^{r}}}+\sum_{{\hat{\psi }}^{{\tilde{d}}_{\tilde{i}}^{r}}}{\tilde{D}}_{\tau^{{\tilde{s}}_{\tilde{i}}^{r}}+{\tilde{\delta }}^{{\tilde{d}}_{\tilde{i}}^{r}}}^{{\hat{\psi }}^{{\tilde{d}}_{\tilde{i}}^{r}}}\right)\right)\geq \sum_{l={\tilde{s}}_{\tilde{i}}^{r}}^{{\tilde{s}}_{\tilde{i}}^{r}+{\tilde{n}}_{\tilde{i}}^{r}-1}{SS}^{r}\left({cf}^{v},{{cr}^{v},cd}_{l}\right),\;\;1\leq \tilde{i}\leq {\tilde{T}}^{r},\;1\leq r\leq {cl}_{s^{v}},$$
  (68) $$\sum_{1\leq \tilde{i}\leq {\tilde{T}}^{r}}\sum_{{\tilde{\delta }}^{{\tilde{d}}_{\tilde{i}}^{r}}}\left(\left(\sum_{\psi^{{\tilde{d}}_{\tilde{i}}^{r}}}{\tilde{D}}_{\tau^{{\tilde{s}}_{\tilde{i}}^{r}}+{\tilde{\delta }}^{{\tilde{d}}_{\tilde{i}}^{r}}}^{\psi^{{\tilde{d}}_{\tilde{i}}^{r}}}+\sum_{{\hat{\psi }}^{{\tilde{d}}_{\tilde{i}}^{r}}}{\tilde{D}}_{\tau^{{\tilde{s}}_{\tilde{i}}^{r}}+{\tilde{\delta }}^{{\tilde{d}}_{\tilde{i}}^{r}}}^{{\hat{\psi }}^{{\tilde{d}}_{\tilde{i}}^{r}}}\right)\right)\geq \sum_{l={tlp}^{v}+1}^{t{lp}^{v}+{\tilde{ps}}^{v}}{SS}^{r}\left({cf}^{v},{{cr}^{v},cd}_{l}\right),\;1\leq \tilde{i}\leq {\tilde{T}}^{r},1\leq r\leq {cl}_{s^{v}},$$
  (69) $${\tilde{s}}_{1}^{r}={tlp}^{v}+1,\;1\leq r\leq {cl}_{s^{v}}$$
  (70) $${\tilde{s}}_{\tilde{i}}^{r}={\tilde{s}}_{\tilde{i}-1}^{r}{+\tilde{n}}_{\tilde{k}-1}^{r},\;\;1<\tilde{i}\leq {\tilde{T}}^{r},$$
  (71) $${\tilde{s}}_{{\tilde{T}}^{r}}^{r}\leq {{tlp}^{v}+\tilde{ps}}^{v},1\leq r\leq {cl}_{s^{v}},$$
  (72) $${\tilde{ps}}^{v}=\sum_{1\leq \tilde{i}\leq {\tilde{T}}^{v}}{\tilde{n}}_{\tilde{i}}^{r},\;1\leq r\leq {cl}_{s^{v}},\;$$
  (73) $$\sum_{l={tlp}^{v}+1}^{t{lp}^{v}+{\tilde{ps}}^{v}}{cd}_{l}\leq {TSL}^{v},\;\;{tlp}^{v}+{\tilde{ps}}^{v}\leq {SC}^{v},$$
  (74) $$\tau^{{\tilde{s}}_{\tilde{i}}^{r}}+{\tilde{\delta }}^{{\tilde{d}}_{\tilde{i}}^{r}}\leq {cpt}_{{\tilde{s}}_{\tilde{i}}^{r}}^{v}<c{pt}_{{SC}^{v}}^{v},\;\;1\leq \tilde{i}\leq {\tilde{T}}^{r},$$
  (75) $${cpt}_{{\tilde{s}}_{\tilde{i}}^{r}-1}^{v}\leq {cpt}_{{\tilde{s}}_{\tilde{i}}^{r}}^{v},\;\;1<\tilde{i}\leq {\tilde{T}}^{r},$$
  (76) $${tbuf}^{\sigma }+\sum_{l={tlp}^{v}+1}^{t{lp}^{v}+{\tilde{ps}}^{v}}\sum_{r=1}^{{cl}^{v}}{SS}^{r}\left({cf}^{v},{{cr}^{v},cd}_{l}\right)\leq {\bar{buf}}^{\sigma },$$
  where ${NMT}^{r}$ represents the sequence of multicast tree nodes allocated to download bandwidth for the r-th encoding layer, and ${\tilde{d}}_{\tilde{i}}^{r}$ denotes the position of the $\tilde{i}$-th node in ${NMT}^{r}$. ${SMT}^{r}$ and ${CMT}^{r}$ sequences, respectively, stand for the video segment indices and the number of video segments downloaded at each node. ${\tilde{T}}^{r}$ is the number of nodes in the sequences ${NMT}^{r}$, ${SMT}^{r}$, and ${CMT}^{r}$. $\psi^{{\tilde{d}}_{\tilde{i}}^{r}}$ and ${\hat{\psi }}^{{\tilde{d}}_{\tilde{i}}^{r}}$ denote the base stations and distributed access point cluster members covering node ${\tilde{d}}_{\tilde{i}}^{r}$ in sequence ${NMT}^{r}$. ${\tilde{D}}_{\tau^{{\tilde{s}}_{\tilde{i}}^{r}}+{\tilde{\delta }}^{{\tilde{d}}_{\tilde{i}}^{r}}}^{\psi^{{\tilde{d}}_{\tilde{i}}^{r}}}$ and ${\tilde{D}}_{\tau^{{\tilde{s}}_{\tilde{i}}^{r}}+{\tilde{\delta }}^{{\tilde{d}}_{\tilde{i}}^{r}}}^{{\hat{\psi }}^{{\tilde{d}}_{\tilde{i}}^{r}}}$ represent the bandwidth allocated by base stations and distributed access point clusters at node ${\tilde{d}}_{\tilde{i}}^{r}$ for non-live video portions. ${\tilde{\delta }}^{{\tilde{d}}_{\tilde{i}}^{r}}$ is the number of consecutive time slots used by node ${\tilde{d}}_{\tilde{k}}^{r}$ for video segment downloads after $\tau^{{\tilde{s}}_{\tilde{i}}^{r}}$.
  This step orchestrates the allocation of bandwidth for downloading additional non-live video segments by leveraging multicast tree structures managed by regional aerial–ground servers. It constructs three sequences—${NMT}^{r}$, ${SMT}^{r}$, and ${CMT}^{r}$—to define which nodes handle which segment layers and in what quantity. The accompanying constraints, as given in Equations (67)–(76), ensure that each node has sufficient bandwidth, segment assignments are sequential and non-overlapping, timing aligns with playback windows, and total buffer capacity is respected. Accordingly, this step ensures efficient, scalable segment preloading across distributed infrastructure while maintaining smooth playback and resource compliance.
Step 8: The regional aerial–ground network management servers responsible for the multicast tree nodes calculated in the previous step are notified to activate the “Non-Live Video Aerial-Ground Network Bandwidth Support Module”. These servers will then determine whether their managed base stations or distributed access point clusters can relay bandwidth for video v via drones or aerial platforms.
Step 9: If the previous step fails to establish a drone or aerial platform relay path to provide bandwidth for the EV user, the system proceeds to Step 11. Otherwise, the following temporary variables are updated:
  (77) $${TSL}^{v}={TSL}^{v}-\sum_{l={tlp}^{v}+1}^{t{lp}^{v}+{\tilde{ps}}^{v}}{cd}_{l},$$
  (78) $${{tbuf}^{\sigma }=tbuf}^{\sigma }+\sum_{l={tlp}^{v}+1}^{t{lp}^{v}+{\tilde{ps}}^{v}}\sum_{r=1}^{{cl}^{v}}{SS}^{r}\left({cf}^{v},{{cr}^{v},cd}_{l}\right),$$
  (79) $$t{lp}^{v}=t{lp}^{v}+{\tilde{ps}}^{v}.$$
  This step handles the system’s response after successfully securing aerial or drone-based bandwidth relays for non-live video delivery. Upon confirmation from Step 8, it updates three key temporary variables to reflect the impact of newly preloaded segments: the remaining playable duration (${TSL}^{v}$) is reduced by the playback time of the newly downloaded segments; the buffer usage (${tbuf}^{\sigma }$) is increased according to the data size of the downloaded segments across all encoding layers; and the playback index ($t{lp}^{v}$) is advanced to indicate the last buffered segment. These updates maintain accurate system state and ensure continuity in subsequent scheduling decisions.
Step 10: If ${TSL}^{v}$ is greater than or equal to the system-defined minimum playback duration for preloaded video segments, the system proceeds to Step 15. Otherwise, it continues to the subsequent step.
Step 11: If video stream v does not contain enhancement layer encoding, the system proceeds directly to Step 13. Otherwise, it continues to the subsequent step.
Step 12: After removing the enhancement layer via Equation (80), the system transitions to Step 3 to proceed.
  (80) $${cl}^{v}=1.$$
  As shown in Equation (80), the system discards all enhancement layers, thereby reducing both the data size and the complexity of the video stream.
Step 13: If the service supports frame rate reduction and the current frame rate has not yet reached the minimum threshold defined by the system, this step adjusts the passenger’s video frame rate as specified below, then proceeds to Step 3 to continue processing.
  (81) $${cf}^{v}=\left\{\begin{array}{cc} {vf}_{f^{v}-1} & if\;f^{v}>1,\\ {vf}_{1} & otherwise \end{array}\right.,$$
  (82) $$f^{v}=f^{v}-1,\;\;if\;f^{v}>1.$$
  By lowering the frame rate using Equation (81) and updating the frame rate index as in Equation (82), the system decreases bandwidth demand while maintaining video continuity, then returns to Step 3 for further processing.
Step 14: When resolution reduction is supported and the current level exceeds the minimum threshold, the system adapts the video resolution per the given equation and re-enters Step 3.
  (83) $${cr}^{v}=\left\{\begin{array}{cc} {vr}_{q^{v}-1} & if\;q^{v}>1,\\ {vr}_{1} & otherwise \end{array}\right.,$$
  (84) $$q^{v}=q^{v}-1,\;\;if\;q^{v}>1.$$
  By adjusting the resolution according to Equation (83) and updating the resolution index in Equation (84), this step reduces bandwidth usage while preserving playback.
Step 15: The base stations, distributed access point clusters, and multicast tree nodes involved in the bandwidth allocation process are notified to download all video portions, and the following variables are updated:
  (85) $${buf}^{\sigma }={tbuf}^{\sigma },$$
  (86) $${lp}^{v}=t{lp}^{v},$$
  (87) $${SL}^{v}={\bar{SL}}^{v}-t{lp}^{v}+{cp}^{v}.$$
  where ${cp}^{v}$ represents the index of the video segment currently being played by the EV.
  This step updates key variables to reflect the current playback and buffering status: ${buf}^{\sigma }$ is set to the updated buffer capacity ${tbuf}^{\sigma }$, ${lp}^{v}$ is updated to the latest last preloaded segment index $t{lp}^{v}$, and the remaining video length ${SL}^{v}$ is recalculated based on the total segments (${\bar{SL}}^{v}$), the updated last preloaded segment, and the current playback position ${cp}^{v}$. This ensures synchronization of system state with ongoing video playback and preloading.
Step 16: This module continues video playback in the background mode until the EV user stops the video playback.
Step 17: Upon arriving at the next intersection, if ${lp}^{v}$ is less than ${SC}^{v}$, the system returns to Step 3 to resume execution.

### 3.4. Emergency/Live Video Aerial-Ground Network Bandwidth Support Module

**Figure 6:**
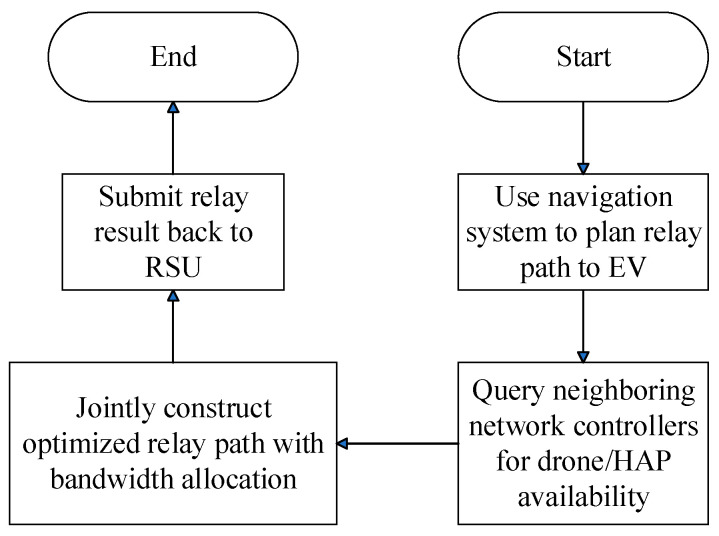
Flowchart of the Emergency/Live Video Aerial-Ground Network Bandwidth Support Module.

As illustrated in Figure 6, when the regional aerial–ground network management server at the location of the multicast tree node designated by the RSU receives a bandwidth request, it activates this module to assist in relaying bandwidth. This module first identifies base stations or distributed access point clusters within its managed area and calculates the joint aerial–ground network relay paths extending toward the EV’s location. This aerial–ground network structure not only fulfills the video bandwidth demands of the EV user but also optimizes overall network utilization by leveraging available wireless bandwidth in uncongested roadways.

This module flows through the following steps:

Step 1: The regional aerial–ground network management server uses navigation software to determine the relay route from its managed base stations or distributed access point clusters to the EV’s location.
Step 2: The server queries nearby regional aerial–ground network management servers for information on aerial platform and drone deployment within their respective coverage areas.
Step 3: In collaboration with the surrounding regional aerial–ground network management servers, a bandwidth relay path is established with the EV requesting bandwidth using the following optimization formulas:
  (88) $$\underset{M^{v}}{arg}\;Min\left|\sum_{m=1}^{M^{v}}\left(\sum_{\psi^{r_{m,1}^{v}}}U_{t_{j}^{v}}^{\psi^{r_{m,1}^{v}}}+\sum_{{\hat{\psi }}^{r_{m,1}^{v}}}U_{t_{j}^{v}}^{{\hat{\psi }}^{r_{m,1}^{v}}}\right)+{dub}_{t_{j}^{v}}\right|,\;\;{if\;dub}_{t_{j}^{v}}<0,$$
  (89) $$\underset{{\tilde{M}}^{v}}{arg}\;Min\left|\sum_{m=1}^{{\tilde{M}}^{v}}\left(\sum_{\psi^{{\tilde{r}}_{m,1}^{v}}}D_{t_{j}^{v}}^{\psi^{{\tilde{r}}_{m,1}^{v}}}+\sum_{{\hat{\psi }}^{{\tilde{r}}_{m,1}^{v}}}D_{t_{j}^{v}}^{{\hat{\psi }}^{{\tilde{r}}_{m,1}^{v}}}\right)+{ddb}_{t_{j}^{v}}\right|,\;\;{if\;ddb}_{t_{j}^{v}}<0$$
  subject to
  (90) $$R_{m}^{v}=\left(r_{m,1}^{v},r_{m,2}^{v},\cdots ,r_{m,\sigma_{m}^{v}-1}^{v},r_{m,\sigma_{m}^{v}}^{v}\right),\;\;\;1\leq m\leq M^{v}$$
  (91) $${\tilde{R}}_{m}^{v}=\left({\tilde{r}}_{m,1}^{v},{\tilde{r}}_{m,2}^{v},\cdots ,{\tilde{r}}_{m,\sigma_{m}^{v}-1}^{v},{\tilde{r}}_{m,{\tilde{\sigma }}_{m}^{v}}^{v}\right),\;\;\;1\leq m\leq {\tilde{M}}^{v},$$
  (92) $$\sum_{m=1}^{M^{v}}\left(\sum_{\psi^{r_{m,1}^{v}}}U_{t_{j}^{v}}^{\psi^{r_{m,1}^{v}}}+\sum_{{\hat{\psi }}^{r_{m,1}^{v}}}U_{t_{j}^{v}}^{{\hat{\psi }}^{r_{m,1}^{v}}}\right)+{dub}_{t_{j}^{v}}\geq 0,\;{1\leq j\leq E^{v},\;\;if\;dub}_{t_{j}^{v}}<0,$$
  (93) $$\sum_{m=1}^{{\tilde{M}}^{v}}\left(\sum_{\psi^{{\tilde{r}}_{m,1}^{v}}}D_{t_{j}^{v}}^{\psi^{{\tilde{r}}_{m,1}^{v}}}+\sum_{{\hat{\psi }}^{{\tilde{r}}_{m,1}^{v}}}D_{t_{j}^{v}}^{{\hat{\psi }}^{{\tilde{r}}_{m,1}^{v}}}\right)+{ddb}_{t_{j}^{v}}\geq 0,\;{1\leq j\leq E^{v},\;\;if\;ddb}_{t_{j}^{v}}<0,$$
  (94) $$r_{m,\sigma_{m}^{v}}^{v}=,\;\;1\leq m\leq M^{v},$$
  (95) $${\tilde{r}}_{m,{\tilde{\sigma }}_{m}^{v}}^{v}=,\;\;1\leq m\leq {\tilde{M}}^{v},$$
  (96) $${urb}_{t_{j}^{v}}^{r_{m,i}^{v},r_{m,i+1}^{v}}\geq {uvb}_{t_{j}^{v}}^{r_{m,i}^{v},r_{m,i+1}^{v}},\;\;1\leq i<\sigma_{m}^{v},\;1\leq m\leq M^{v},\;1\leq j\leq E^{v},\;\;{if\;dub}_{t_{j}^{v}}<0,$$
  (97) $${drb}_{t_{j}^{v}}^{{\tilde{r}}_{m,i}^{v},{\tilde{r}}_{m,i+1}^{v}}\geq {dvb}_{t_{j}^{v}}^{{\tilde{r}}_{m,i}^{v},{\tilde{r}}_{m,i+1}^{v}},\;\;1\leq i<{\tilde{\sigma }}_{m}^{v},\;1\leq m\leq {\tilde{M}}^{v},\;1\leq j\leq E^{v},\;\;{if\;ddb}_{t_{j}^{v}}<0,$$
  where $R_{m}^{v}$ and ${\tilde{R}}_{m}^{v}$ represent the *m*-th uplink and downlink aerial–ground relay paths that can support v’s bandwidth demand. $M^{v}$ and ${\tilde{M}}^{v}$ are the numbers of uplink and downlink relay paths, while $\sigma_{m}^{v}$ and ${\tilde{\sigma }}_{m}^{v}$ denote the numbers of nodes along the *m*-th uplink and downlink relay paths, respectively. $r_{m,1}^{v}$ and ${\tilde{r}}_{m,1}^{v}$ represent the source base stations or distributed access point clusters for the uplink and downlink aerial–ground relay paths, respectively, while $r_{m,\sigma_{m}^{v}}^{v}$ is the EV *σ* at the end of the relay path. ${urb}_{t_{j}^{v}}^{r_{m,i}^{v},r_{m,i+1}^{v}}$ and ${drb}_{t_{j}^{v}}^{{\tilde{r}}_{m,i}^{v},{\tilde{r}}_{m,i+1}^{v}}$ represent the remaining available bandwidth from $r_{m,i+1}^{v}$ and from ${\tilde{r}}_{m,i}^{v}$ to ${\tilde{r}}_{m,i+1}^{v}$, respectively, while ${uvb}_{t_{j}^{v}}^{r_{m,i}^{v},r_{m,i+1}^{v}}$ and ${dvb}_{t_{j}^{v}}^{{\tilde{r}}_{m,i}^{v},{\tilde{r}}_{m,i+1}^{v}}$ denote the required bandwidths for uplink and downlink transmission. Unless otherwise noted, all parameters are as defined in Section 3.2.
  Equations (88) and (89) minimize the total bandwidth load across multiple relay paths, balancing the demands and available capacity. Paths $R_{m}^{v}$ and ${\tilde{R}}_{m}^{v}$, defined in Equations (90) and (91), represent sequences of nodes—from base stations or access points to the EV—that must maintain sufficient bandwidth, as specified in Equations (92)–(97). The constraints ensure that each path terminates at the EV, uplink and downlink bandwidth requirements do not exceed available resources, and overall allocation efficiently supports the EV’s streaming demands. Step 4 involves sending the finalized bandwidth relay setup results to the RSU.
Step 4: The bandwidth relay setup results are sent to the RSU.

### 3.5. Non-Live Video Aerial-Ground Network Bandwidth Support Module

**Figure 7:**
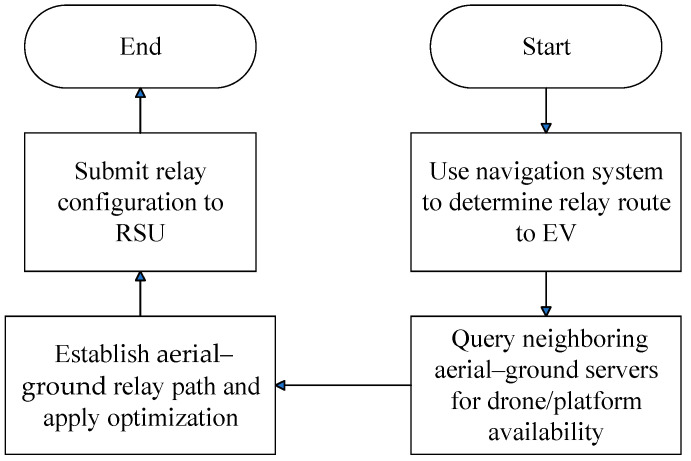
Flowchart of the Non-Live Video Aerial–Ground Network Bandwidth Support Module.

As illustrated in Figure 7, when the regional aerial–ground network management server—located at the multicast tree node specified by the RSU—receives a bandwidth request, it activates this module to enable the advance retrieval of video portions. This module then identifies candidate base stations or distributed access point clusters within its coverage area and computes suitable aerial–ground relay paths for delivering the video segments from its managed region to the current location of the EV along its travel route.

This module flows through the following steps:

Step 1: The regional aerial–ground network management server uses navigation software to determine the relay route from its managed base stations or distributed access point clusters to the EV’s location.
Step 2: The server queries neighboring regional aerial–ground network management servers for aerial platform and drone deployment information within their respective coverage areas.
Step 3: In collaboration with the surrounding regional aerial–ground network management servers, an aerial–ground network relay path is established to pre-download video segments for the EV as follows:
  (98) $$\underset{{\hat{M}}^{v}}{arg}\;Min\left|\sum_{m=1}^{{\hat{M}}^{v,\tilde{j}}}\left(\sum_{\psi^{{\hat{r}}_{m,1}^{v,\tilde{j}}}}D_{\tau_{{\tilde{s}}_{\tilde{j}}^{v}}+{\tilde{\delta }}^{{\tilde{d}}_{\tilde{j}}^{v}}}^{\psi^{{\hat{r}}_{m,1}^{v,\tilde{j}}}}+\sum_{{\hat{\psi }}^{{\tilde{r}}_{m,1}^{v}}}D_{\tau_{{\tilde{s}}_{\tilde{j}}^{v}}+{\tilde{\delta }}^{{\tilde{d}}_{\tilde{j}}^{v}}}^{{\hat{\psi }}^{{\hat{r}}_{m,1}^{v,\tilde{j}}}}\right)-\sum_{l={\tilde{s}}_{\tilde{j}}^{v}}^{{\tilde{n}}_{\tilde{j}}^{v}+{\tilde{s}}_{\tilde{j}}^{v}-1}\sum_{r=1}^{{cl}_{l}}{SS}^{r}\left({cf}_{l},{{cr}_{l},cd}_{l}\right)\right|,$$
  subject to:
  (99) $${\hat{R}}_{m}^{v,\tilde{j}}=\left({\hat{r}}_{m,1}^{v,\tilde{j}},{\hat{r}}_{m,2}^{v,\tilde{j}},\cdots ,{\hat{r}}_{m,{\hat{\sigma }}_{m}^{v}-1}^{v,\tilde{j}},{\hat{r}}_{m,{\hat{\sigma }}_{m}^{v}}^{v,\tilde{j}}\right),\;\;1\leq m\leq {\tilde{M}}^{v,\tilde{j}},$$
  (100) $$\sum_{m=1}^{{\hat{M}}^{v,\tilde{j}}}\left(\sum_{\psi^{{\hat{r}}_{m,1}^{v,\tilde{j}}}}D_{\tau_{{\tilde{s}}_{\tilde{j}}^{v}}+{\tilde{\delta }}^{{\tilde{d}}_{\tilde{j}}^{v}}}^{\psi^{{\hat{r}}_{m,1}^{v,\tilde{j}}}}+\sum_{{\hat{\psi }}^{{\tilde{r}}_{m,1}^{v}}}D_{\tau_{{\tilde{s}}_{\tilde{j}}^{v}}+{\tilde{\delta }}^{{\tilde{d}}_{\tilde{j}}^{v}}}^{{\hat{\psi }}^{{\hat{r}}_{m,1}^{v,\tilde{j}}}}\right)\geq \sum_{l={\tilde{s}}_{\tilde{j}}^{v}}^{{\tilde{n}}_{\tilde{j}}^{v}+{\tilde{s}}_{\tilde{j}}^{v}-1}\sum_{r=1}^{{cl}_{l}}{SS}^{r}\left({cf}_{l},{{cr}_{l},cd}_{l}\right)$$
  (101) $${\hat{r}}_{m,{\hat{\chi }}_{m}^{v}}^{v,\tilde{j}}=\sigma ,1\leq m\leq {\tilde{M}}^{v,\tilde{j}},$$
  (102) $${drb}_{\tau_{{\tilde{s}}_{\tilde{j}}^{v}}}^{{\hat{r}}_{m,i}^{v,\tilde{j}},{\hat{r}}_{m,i+1}^{v,\tilde{j}}}\geq {dvb}_{\tau_{{\tilde{s}}_{\tilde{j}}^{v}}}^{{\hat{r}}_{m,i}^{v,\tilde{j}},{\hat{r}}_{m,i+1}^{v,\tilde{j}}},\;\;1\leq i<{\hat{\chi }}_{m}^{v},\;1\leq m\leq {\tilde{M}}^{v,\tilde{j}},$$
  where ${\hat{R}}_{m}^{v,\tilde{j}}$ represents the *m*-th aerial–ground network relay path that supports the bandwidth transfer for video v from multicast tree node ${\tilde{d}}_{\tilde{j}}^{v}$. ${\tilde{M}}^{v,\tilde{j}}$ is the number of aerial–ground relay paths for video segment transfer, while ${\hat{\chi }}_{m}^{v}$ denotes the number of nodes along the *m*-th aerial–ground relay path. ${\hat{r}}_{m,1}^{v,\tilde{j}}$ stands for the source base station or distributed access point cluster for the aerial–ground network relay path. ${drb}_{\tau_{{\tilde{s}}_{\tilde{j}}^{v}}}^{{\hat{r}}_{m,i}^{v,\tilde{j}},{\hat{r}}_{m,i+1}^{v,\tilde{j}}}$ represents, at time $\tau_{{\tilde{s}}_{\tilde{j}}^{v}}$, the remaining bandwidth for downloading from node ${\hat{r}}_{m,i}^{v,\tilde{j}}$ to node ${\hat{r}}_{m,i+1}^{v,\tilde{j}}$, and ${dvb}_{t_{j}^{v}}^{{\hat{r}}_{m,i}^{v,\tilde{j}},{\hat{r}}_{m,i+1}^{v,\tilde{j}}}$ denotes, at time $\tau_{{\tilde{s}}_{\tilde{j}}^{v}}$, the total bandwidth required for downloading video segments from node ${\hat{r}}_{m,i}^{v,\tilde{j}}$ to node ${\hat{r}}_{m,i+1}^{v,\tilde{j}}$. Unless otherwise noted, all parameters are as defined in Section 3.3.
  Equation (98) formulates an optimization problem that minimizes the total weighted difference between the bandwidth used to download video segments over multiple aerial–ground relay paths and the sizes of the pre-fetched video segments, balancing network load and caching efficiency. Each relay path ${\hat{R}}_{m}^{v,\tilde{j}}$, defined in Equation (99), is a sequence of nodes—from source base stations or access point clusters to the EV—that transmit video data from multicast tree nodes to the EV. Constraint (100) ensures that the combined bandwidth provided by these relay paths meets or exceeds the demand needed to download all required video segments for smooth playback. Equation (101) guarantees that each relay path ends at the EV’s current location. Finally, inequality (102) enforces that the available bandwidth on each link along the relay path at time $\tau_{{\tilde{s}}_{\tilde{j}}^{v}}$ is sufficient to support the required video download rate, preventing network congestion or bottlenecks during preloading.
Step 4: The finalized bandwidth relay settings are submitted to the RSU.

### 3.6. Decision Metrics, Context Parameters, and Optimization Framework

To improve reproducibility and transparency, this section summarizes the main decision metrics, context parameters, and optimization strategies used throughout the proposed system.

The system relies on several decision variables. Bandwidth allocation decisions are triggered by application class (emergency, live, or non-live), with shortfall metrics defined in Equations (19)–(22) indicating when reallocation is necessary. Candidate multicast relay nodes are ranked based on current uplink or downlink capacity, as defined in Equations (27)–(30), and a greedy sorting heuristic (i.e., selecting the locally optimal option at each step) is used for selection. If network resources remain constrained, video quality is adaptively managed by reducing frame rate (Equations (43)–(45), (81) and (82)) or resolution (Equations (46)–(48), (83) and (84)), or by discarding enhancement layers (Equation (80)) while ensuring base layer-only decoding (i.e., delivering minimum-quality video that remains playable).

The system also integrates several context-aware parameters. These include the EV’s origin, destination, and estimated route, derived from GPS data and third-party navigation APIs such as Google Maps. Traffic forecasts and congestion detection rely on both historical patterns and live updates. Service characteristics such as stream type, encoding structure (e.g., number of SVC layers), resolution, and frame rate are all considered. Additionally, real-time bandwidth availability at base stations and access point clusters, along with the EV’s local buffer and playback status, are continuously monitored.

The optimization process is modular and lightweight. Bandwidth is reserved in advance using route forecasts and time-of-arrival estimates (Equations (3)–(7)). When emergency or live video demands exceed available resources, the system first reallocates bandwidth from non-live services (Equations (23)–(26)), then from lower-priority live streams (Equations (41) and (42)), and finally invokes aerial relay paths through regional servers using a constrained optimization framework (Equations (88)–(97)). Relay nodes are selected based on a sorted bandwidth ranking (i.e., prioritized list based on link capacity) and updated iteratively (Equations (27)–(38)). The system prioritizes base layer delivery to preserve minimum service quality, applying adaptive fallback mechanisms if enhancement layers cannot be supported.

These mechanisms are aligned with the flowcharts and formulations presented in Section 3.1, Section 3.2, Section 3.3, Section 3.4 and Section 3.5 and are designed to ensure reproducibility and system transparency.

Section 4.1 will demonstrate the framework’s real-time feasibility through computational complexity analysis and empirical benchmarks.

## 4. Simulation Results and Analysis

We performed simulations based on the architecture and algorithms described earlier, assuming all network devices are equipped with interoperable communication interfaces to ensure seamless connectivity across different network segments. Since the technologies under study are still evolving, we relied on quantitative simulations to evaluate the performance of the proposed framework under realistic conditions.

**Figure 8:**
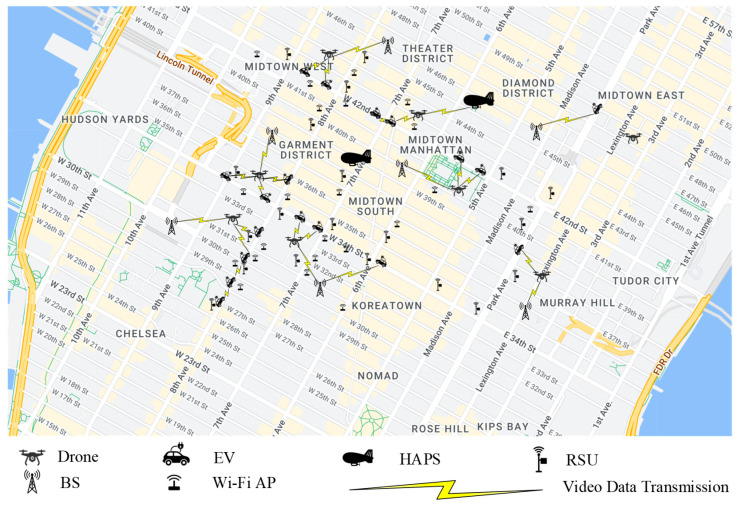
Illustration of aerial-assisted vehicular network over a Midtown Manhattan urban grid.

Our simulation environment specifically models Midtown Manhattan, New York City, using real-world vehicular traffic data [31] to emulate mobility patterns. The EV density (300–500 EVs/km^2^) reflects peak-hour conditions in dense urban cores, where EVs represent ∼15–20% of total traffic (2000–3500 EVs/km^2^ [32,33]), aligning with near-future adoption projections [34]. EV mobility follows a Manhattan grid model [35], characterized by constrained movement along orthogonal urban streets and intersections, with speeds ranging from 5 to 15 m per second. Trip origins and destinations are randomized based on a uniform distribution to reflect actual usage patterns. An illustrative Manhattan grid map (Figure 8) is provided to accurately represent the urban road topology and traffic dynamics. Each simulation scenario covers a 24-h period segmented into 1-h intervals.

The aerial infrastructure consists of drones operating at altitudes between 100 and 300 m with speeds up to 20 m/s, dynamically assisting congested EV clusters, and two HAPs positioned at approximately 20 km altitude providing wide-area coverage. Each EV generates 1–10 Mbps of typical load (with bursts up to 309 Mbps for 360° video [36,37]), matching empirical measurements of UHD/360° streaming demands. Ground base stations offer 1–2 Gbps downlink and 200–500 Mbps uplink capacities per km^2^, ensuring ∼60–80% utilization headroom for 6G mmWave/THz variability [38]. Drones and HAPs serve as supplementary relays, achieving multi-gigabit throughput via mmWave, THz, or FSO links using directional beamforming.

When visibility deteriorates due to dense fog, the system automatically switches from FSO to RF transmission, based on hourly weather report data [39]. EVs primarily access the network via sub-6 GHz and VLC technologies, while mmWave and THz links are utilized under line-of-sight conditions. This parameter selection ensures (1) realistic urban EV penetration, (2) scalability under peak infotainment demands, and (3) compliance with 6G operational thresholds. The comprehensive setup supports detailed evaluation of aerial–ground integrated vehicular networks in dense urban environments.

Table 2 summarizes the transmission ranges and maximum data rates of the wireless communication technologies utilized in our simulations, covering both ground-based and aerial network systems. Table 3 presents the bandwidth requirements for 2D and 360-degree videos. These specifications were obtained through our transmission framework and simulation studies employing SHVC encoding, with total bandwidth consumption computed by aggregating the base layer and enhancement layer requirements [40,41].

In this work, emergency videos are treated as 360-degree video calls, as emergency scenarios, such as remote surgeries, often require multi-angle viewing or real-time demonstrations. The video quality is set to the highest level, with no downgrades allowed. This approach ensures that critical details remain clear, reducing the risks of image blurring or latency that could lead to serious safety concerns.

To benchmark the proposed framework, we compare it with the state-of-the-art method introduced by He et al. [1], hereafter referred to as the baseline. This method represents a typical drone–EV collaboration approach within aerial–ground integrated vehicular networks and serves as a robust reference for assessing improvements in bandwidth allocation efficiency and service differentiation under high-load conditions.

**Figure 9:**
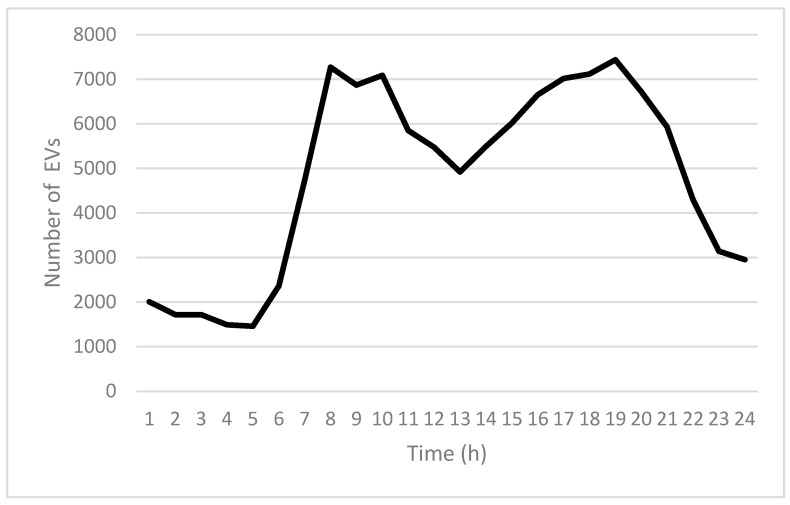
Hourly variation in EV traffic volume over a 24-h period.

Figure 9 illustrates the hourly fluctuations in EV count throughout a typical day. The y-axis indicates the number of EVs, while the x-axis represents the time of day, divided into hourly intervals.

During the early morning period (12 AM–5 AM), Figure 9 indicates that EV numbers maintain a stable range of 1400 to 2000. This interval typically corresponds to off-peak traffic periods, reflecting that most trips have either ended or have not yet commenced. Starting from 6 AM, EV numbers begin to rise sharply, closely linked to the gradual increase in commuter activity as people start their daily routines. This upward trend peaks at around 8 AM, reaching approximately 7267 EVs, signifying a primary morning peak period associated with commuting and daily transportation.

After the 8 AM peak, EV volume slightly declines but remains at a relatively high and stable level (approximately between 5000 and 7000 EVs) from 11 AM to 3 PM. This steady state indicates more evenly distributed EV usage during midday hours, typically reflecting daytime travel patterns without significant fluctuations. Subsequently, from 3 PM onwards, EV numbers begin to rise again, reaching the highest daily peak at around 7 PM, with approximately 7436 EVs. This evening peak likely reflects increased commuter travel as individuals return home or participate in evening activities.

Following the evening peak (8 PM–12 AM), the number of EVs gradually decreases, dropping to around 3000 EVs by midnight. This decline illustrates reduced travel demand and EV activity during night-time hours. Overall, the daily EV volume exhibits a clear bimodal pattern, demonstrating distinct morning and evening peaks, consistent with typical commuter and passenger travel demand patterns.

**Figure 10:**
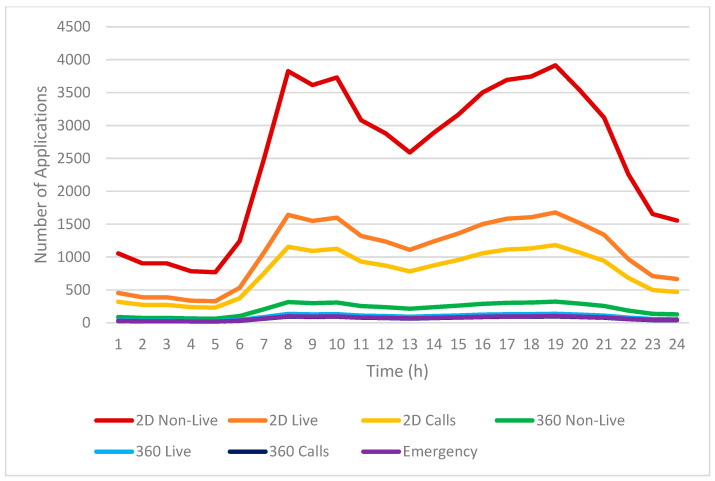
Temporal dynamics of EV passenger-initiated infotainment service requests.

Figure 10 shows the variation in infotainment service usage initiated by EV passengers across different hours of a typical day. The y-axis displays the count of initiated sessions, with the x-axis showing hourly time increments.

As seen in Figure 10, infotainment service usage remains relatively low during early morning hours (from midnight to around 5 AM), corresponding with fewer EVs being active on the road. During this off-peak period, demand for entertainment and communication applications is minimal, likely due to most users being inactive or at rest.

Starting from approximately 6 AM, there is a noticeable increase in infotainment service usage, closely aligned with the rising number of EVs as commuters begin their morning journeys. The usage of infotainment services quickly escalates and reaches the first significant peak at around 8 AM. This morning peak reflects a high demand for multimedia content, such as non-live video and video calls, as passengers seek entertainment or utilize communication tools during commutes.

Following the morning peak, the usage of infotainment services slightly decreases yet remains at a stable and relatively high level throughout the midday hours (approximately from 10 AM to 3 PM). After this stable period, usage begins to rise again from 3 PM onward, coinciding with the increase in EV activity as people commence their evening commutes. The second peak of infotainment service usage occurs around 6 PM, mirroring the patterns observed in the morning peak and reinforcing the correlation between commuting times and application usage.

Among the various infotainment services, 2D non-live video services exhibit the highest usage rates throughout the day, significantly outperforming other applications. EV passengers overwhelmingly choose 2D non-live video for its viewer-centric experience—the freedom to watch content on their own schedule, with complete control over playback, coupled with access to vast media libraries.

Live infotainment services demonstrate significantly lower adoption rates than non-live services. This disparity stems from their inherent requirement for real-time engagement, which conflicts with the irregular schedules of most users. Within live services, the usage of 360° multimedia content (including 360° live videos and 360° video calls) remains comparatively low. The limited adoption is primarily due to the greater complexity involved in producing such immersive content, coupled with higher demands for network bandwidth and stable connectivity—requirements that may not be consistently satisfied during vehicular travel.

Overall, the patterns depicted in Figure 10 underscore a clear correlation between EV usage fluctuations and infotainment service demand, highlighting important implications for bandwidth allocation and network planning in vehicular environments.

**Figure 11:**
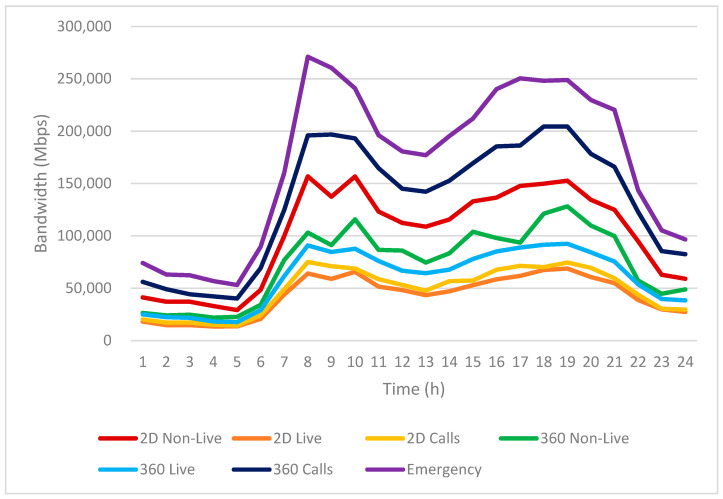
Hourly estimated bandwidth demands for infotainment services in EVs.

Based on the infotainment service usage patterns shown in Figure 10, we further analyze the resulting bandwidth requirements in Figure 11.

Figure 11 illustrates the hourly estimated bandwidth requirements for various infotainment services used by EV passengers over a 24-h period. The vertical axis represents the expected bandwidth in Mbps, while the horizontal axis indicates the time of day, segmented into hourly intervals.

As shown in Figure 11, bandwidth demand remains relatively low during the early morning period, specifically between 12 AM and 5 AM. This period aligns with lower usage rates of infotainment services, corresponding with fewer EVs on the road. Starting from approximately 6 AM, the bandwidth requirements for all infotainment services significantly rise, reflecting increased EV passenger activity during morning commuting hours. The bandwidth usage reaches its first peak around 8 AM, highlighting intense consumption of multimedia content, especially live and emergency applications, during peak commuting times.

Throughout the midday hours (approximately 10 AM to 3 PM), bandwidth usage remains relatively stable yet still at elevated levels compared to early morning hours. Beginning from around 3 PM, there is another noticeable increase in bandwidth demand, coinciding with passengers’ evening commutes. Bandwidth usage reaches its second daily peak between 6 PM and 8 PM, mirroring patterns observed in the morning peak, and subsequently declines gradually toward midnight.

Emergency applications demonstrate the most substantial bandwidth requirements among all infotainment services. This elevated demand results from their critical operational needs, including high-resolution 360° video streaming, ultra-low latency communication, and reliable bidirectional data transfer for real-time emergency response coordination.

Following emergency services, 360° call applications exhibit the next highest bandwidth consumption, surpassing every other 360° and 2D category. The significant resource requirements for 360° media stem from the need to process and transmit spherical video data at high frame rates and resolutions.

An interesting dichotomy emerges between live and non-live content. While non-live video applications (including both 2D and 360° formats) benefit from advanced compression algorithms like VVC (Versatile Video Coding), their live counterparts demand substantially greater bandwidth due to real-time encoding constraints and the necessity for uninterrupted bidirectional data flow.

The most resource-intensive application is unequivocally 360° live video streaming, which combines the challenges of spherical video processing, multi-user synchronization, and strict quality-of-service requirements. This application category consistently dominates network resource allocation during peak usage periods.

Overall, the hourly patterns depicted in Figure 11 underline critical insights for network planning, emphasizing the necessity for dynamic bandwidth allocation strategies in heterogeneous V2X networks to accommodate varying multimedia demands and ensure consistent service quality for EV passengers.

**Figure 12:**
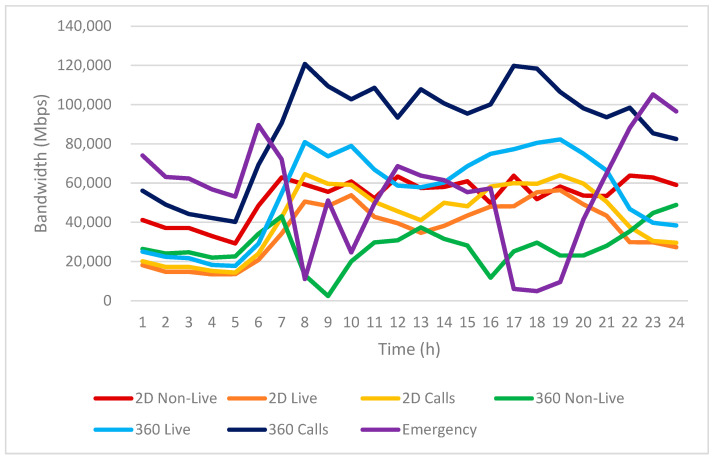
Temporal Bandwidth Allocation for EV Infotainment in the Baseline.

Figure 12 illustrates the temporal distribution of bandwidth allocated to infotainment services under the baseline scheme [1], which serves as a benchmark for evaluating the performance of the proposed resource management framework. As shown in the figure, the baseline framework maintains relatively stable bandwidth provisioning during off-peak hours (00:00–05:00), when vehicular density and infotainment demand remain low. This stability enables most services to operate without noticeable degradation in user experience. The consistent performance during these periods reflects the baseline system’s capability to handle low-load conditions without significant coordination overhead.

However, beginning around 06:00, the network faces a steep increase in bandwidth demand, driven by a surge in vehicular activity and multimedia usage, particularly during the morning commuting window. This sharp rise in service requests, especially for bandwidth-intensive applications, leads to pronounced fluctuations and frequent instances of under-provisioning. The baseline system, which lacks adaptive coordination mechanisms and operates without aerial support or dynamic scheduling, struggles to maintain consistent network performance under such rapidly changing conditions.

Throughout the daytime period (08:00–20:00), the allocated bandwidth remains inconsistent across service categories. In particular, emergency services and 360° infotainment applications (e.g., immersive video calls and live streaming) suffer from frequent bandwidth dips, which correlate with periods of peak network load. These fluctuations highlight the baseline scheme’s limited capacity to differentiate services or prioritize mission-critical or delay-sensitive traffic. The absence of centralized control or collaborative management contributes to inefficient resource utilization, resulting in degraded user-perceived media performance, including service interruptions, video buffering, and unstable content delivery.

After 20:00, the decline in EV activity leads to a gradual stabilization of bandwidth allocation. By late evening, the system regains its ability to meet service demands, and user-perceived performance returns to acceptable levels.

In summary, Figure 12 underscores the operational constraints of the baseline scheme, which relies on static allocation and lacks cross-layer coordination. The observed inefficiencies—particularly during high-demand periods—reinforce the advantages of the proposed approach in delivering more stable, efficient, and user-satisfactory multimedia services.

**Figure 13:**
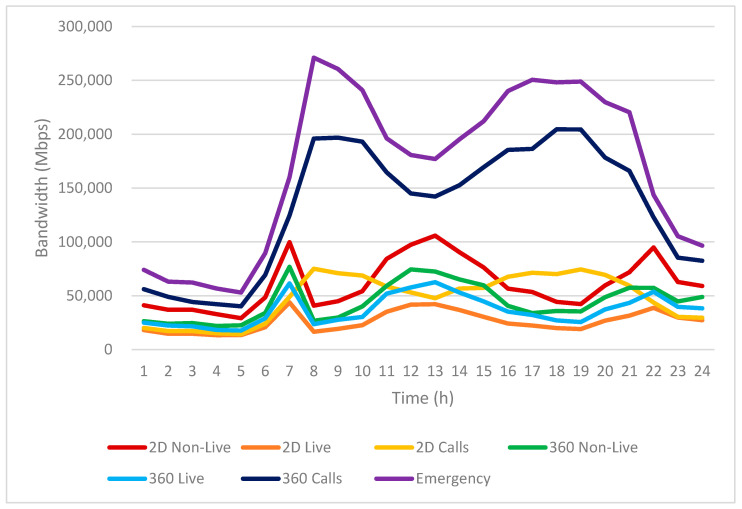
Temporal bandwidth allocation for EV infotainment under the proposed framework.

Figure 13 demonstrates the proposed resource assignment patterns, showcasing our mechanism’s efficacy in optimizing multimedia resource distribution for vehicular infotainment services. During off-peak hours (0 AM–5 AM), the system maintains consistently sufficient bandwidth capacity, where diminished EV traffic corresponds with reduced multimedia demand, enabling reliable service provisioning.

Starting from 6 AM, as EV passenger activity increases, bandwidth demand escalates significantly. However, in contrast to the situation before the implementation of the proposed mechanism, a marked improvement by effectively managing and prioritizing bandwidth resources is observed. Notably, at around 8 AM—peak commuting hours—the proposed mechanism effectively allocates higher bandwidth to critical applications, particularly emergency and live services (e.g., 360° video calls). This efficient prioritization is a direct result of the algorithm’s “Emergency/Live Video Resource Harvesting Module”, ensuring stable and high-quality multimedia experiences even during peak demand periods.

Throughout the midday period (10 AM to 5 PM), the proposed work continues to efficiently manage bandwidth resources, allocating more consistent and stable bandwidth levels to each application compared to the scenario in conventional vehicular networks. Consequently, the stability in bandwidth allocation significantly reduces service degradation issues, such as buffering or dropped connections, thus improving overall user experience.

Additionally, the implementation of the “Non-Live Video Air-Ground Network Bandwidth Support Module” effectively balances bandwidth distribution across EVs, maintaining consistent multimedia performance across different geographic areas and EV populations. This dynamic reallocation ensures that bandwidth-intensive applications, particularly emergency services and 360° multimedia content, receive the necessary resources throughout the day.

During evening hours (5 PM to 10 PM), the proposed mechanism maintains stable and sufficient bandwidth allocation, responding effectively to increased demand during the evening commute. Unlike the pre-adjustment scenario, bandwidth allocations remain consistently high, thus ensuring reliable infotainment service.

In summary, Figure 13 demonstrates the substantial improvements achieved by implementing the proposed bandwidth management framework. The enhanced bandwidth allocation strategy significantly stabilizes infotainment service performance, meets critical demands effectively, and markedly improves user experiences, even in highly dynamic and densely populated EV environments.

**Figure 14:**
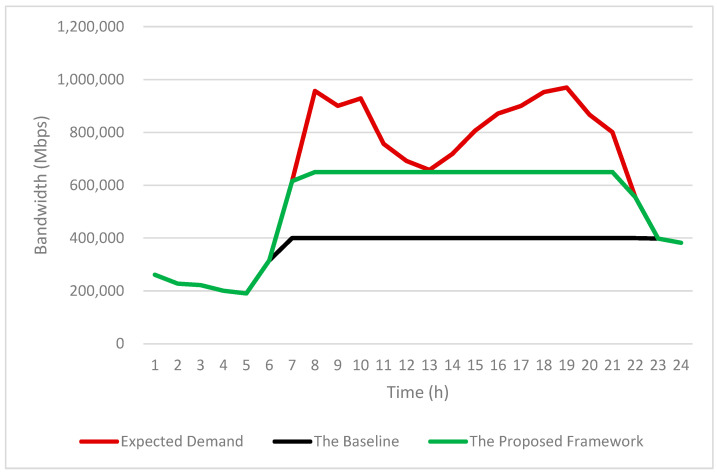
Expected vs. allocated bandwidth for the baseline and the proposed framework.

To clearly illustrate the improvements achieved, Figure 14 directly compares the bandwidth allocation results before and after implementing our proposed mechanism.

Figure 14 provides a comparative view of the total bandwidth demand from infotainment services versus the bandwidth allocations before and after applying the proposed mechanism. The y-axis represents the bandwidth in Mbps, while the x-axis corresponds to hourly intervals across the entire day.

As shown in Figure 14, during the early hours of the day (approximately 0 AM–5 AM), the projected bandwidth demand remains modest. In this time window, the allocated bandwidth, both before and after the implementation of the proposed mechanism, aligns closely with the expected demand, suggesting that the existing network resources are adequate to satisfy user requirements without necessitating additional bandwidth management interventions.

However, beginning at approximately 7 AM, there is a noticeable rise in expected bandwidth demand, reaching its peak during the morning commuting hours (8 AM to 10 AM) and again during evening hours (from around 5 PM to 8 PM). Before the implementation of the proposed mechanism, the allocated bandwidth was capped at a fixed limit (approximately 400,000 Mbps), resulting in a substantial gap between allocated bandwidth and actual bandwidth requirements. Consequently, users experienced degraded multimedia performance, particularly during these peak periods.

After applying the proposed framework, as depicted by the green curve in Figure 14, allocated bandwidth increases significantly during critical peak hours, closely aligning with the actual user demand. Specifically, the proposed work adjusts the bandwidth dynamically from around 7 AM onward, maintaining a higher bandwidth allocation (approximately 650,000 Mbps) throughout most of the peak daytime period. This adjustment substantially reduces the bandwidth gap compared to the previous fixed allocation approach, significantly enhancing the multimedia experience by providing sufficient resources during periods of high demand.

Notably, during off-peak and late-night hours (after 10 PM), both schemes return to similar lower levels, aligning again with the expected bandwidth demands.

In summary, Figure 14 clearly demonstrates the effectiveness of the proposed mechanism in addressing bandwidth shortages during peak hours. By dynamically adjusting resource allocation according to real-time demand, the proposed work achieves more efficient bandwidth utilization, reducing network congestion and greatly improving infotainment service quality for EV users throughout the day.

**Figure 15:**
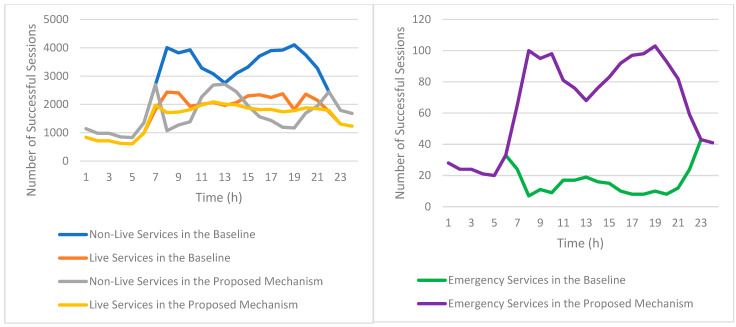
Comparison of bandwidth-compliant applications: proposed framework vs. baseline.

Figure 15 presents a comparative analysis of the number of bandwidth-compliant infotainment service sessions supported by the proposed framework versus the baseline. The analysis categorizes applications into three types: non-live, live, and emergency services. Emergency services are treated separately due to their distinct bandwidth compliance characteristics. The vertical axis shows the number of successfully delivered sessions, while the horizontal axis represents hourly intervals over a 24-h period.

From midnight to 6 AM, both frameworks perform similarly across all categories, reflecting low demand and ample bandwidth availability. This indicates that during off-peak hours, both approaches sufficiently support infotainment services.

Performance differences become evident starting around 7 AM, coinciding with increased network load during the morning commute. The baseline scheme exhibits two key limitations: persistently low success rates for emergency services, highlighting a lack of prioritization for critical traffic, and fluctuating delivery rates for live and non-live applications, causing instability and degraded user experience during peak times.

In contrast, the proposed framework achieves notable improvements, particularly for live and emergency applications. Emergency service delivery is significantly enhanced, demonstrating effective prioritization and efficient bandwidth management through dynamic V2V and air–ground relay mechanisms. Additionally, the framework maintains more consistent service levels for live applications throughout the day.

Although the number of successfully delivered non-live sessions occasionally decreases under the proposed framework, this trade-off is intentional to prioritize emergency applications, thereby optimizing overall resource utilization.

Overall, Figure 15 clearly demonstrates the proposed framework’s superior capability to meet the diverse and dynamic performance demands of vehicular infotainment systems, surpassing the limitations of static bandwidth allocation by dynamically adjusting resources based on real-time application requirements and network conditions. This results in improved reliability, responsiveness, and user satisfaction across varying traffic scenarios.

### 4.1. Computational Complexity Analysis

This section presents a high-level evaluation of the computational complexity of the proposed algorithm, focusing on its scalability across key operational variables. Let *N* denote the number of waypoints or intersections in the EV’s route, *M* the number of multicast tree nodes, *L* the number of video segments, *K* the number of encoding layers, *T* the number of time slots in the planning window, *B* the number of base stations in range, and *R* the number of candidate relay paths.

The adaptive video resource manager (Section 3.1) computes route and congestion forecasts in linear time with respect to *N,* i.e., O(N). In the emergency and live video resource harvesting module (Section 3.2), bandwidth gap computation scales as O(B·K·T), and the dominating step is multicast-node sorting at O(M·logM). The non-live video scheduling module (Section 3.3) involves segment-to-node and segment-to-layer mapping (i.e., assigning video chunks to network nodes and quality layers) with complexity O(L·M+L·K). Relay path planning for both live and non-live cases (Section 3.4 and Section 3.5) examines *R* candidates, each of up to *N* hops, yielding O(R·N). Consequently, the overall worst-case complexity is summarized as O(M·logM+L·M+R·N).

This reflects the dominant operations: multicast sorting, segment scheduling, and multi-hop relay planning. As *M*, *L*, and *R* are typically bounded (tens to hundreds) in practical deployments, the algorithm remains efficient and suitable for real-time use.

#### 4.1.1. Comparison to the Baseline

In the baseline, a closed-form expression is derived for the optimal drone–base station altitude, and the sum-rate maximization problem is decoupled into joint power control and spectrum allocation subproblems. The overall solution operates with polynomial-time complexity. Specifically, the optimal altitude is determined in constant time, i.e., O(1), while the sum-rate optimization—primarily involving power control and spectrum sharing across *D* channels or drones—is solvable in polynomial time, likely ranging from $O(D^{2})$ to $O(D^{3})$, depending on matrix inversion or convex optimization steps per iteration.

#### 4.1.2. Comparative Summary

Table 4 summarizes the computational complexity characteristics of the proposed algorithm and the one presented by [1]. While both algorithms are designed to support real-time aerial-assisted vehicular services, they differ significantly in their structure and scalability. This analysis confirms that both algorithms offer polynomial-time solutions appropriate for deployment in real-time networked vehicular environments, though they take different approaches in balancing scalability, control granularity, and computational cost.

This analysis confirms that both algorithms offer polynomial-time solutions appropriate for deployment in real-time networked vehicular environments, though they take different approaches in balancing scalability, control granularity, and computational cost.

#### 4.1.3. Communication and Coordination Overhead

Beyond computational complexity, the proposed architecture introduces coordination overhead associated with signaling between EVs, RSUs, base stations, and drones. Bandwidth reservation for upcoming intersections (as in Equations (3)–(7)) and segment request propagation (Equations (54)–(59)) involve lightweight message exchanges, typically once per planning interval (e.g., every few seconds). Uplink/downlink availability at multicast nodes is disseminated periodically (Equations (27)–(30)), enabling real-time relay decisions with limited signaling cost. Since most logic resides in the RSUs or cloud-side edge servers, EVs offload complexity, incurring only low-volume control traffic. The system leverages existing V2X protocols (e.g., IEEE 802.11p, LTE-V2X) for signaling, ensuring compatibility with current vehicular networks and minimizing overhead per control cycle.

In contrast, the baseline architecture proposed by [1] requires more frequent channel feedback from EVs to drones for real-time CSI updates and resource allocation. Each drone performs centralized optimization based on global or semi-global knowledge, resulting in more intensive signaling per decision cycle. Additionally, drones must coordinate among themselves to avoid spectrum conflict and maintain sum-rate fairness. As the number of drones and EVs grows, the signaling load scales quadratically in the worst case, which could introduce bottlenecks in dense deployments.

## 5. Conclusions

This study introduces a novel aerial–ground vehicular network architecture designed to enhance multimedia service delivery in urban EV environments. The proposed system integrates terrestrial infrastructure with aerial platforms—namely drones and HAPs—leveraging advanced wireless technologies such as VLC, mmWave, and THz communication. The multi-layered design enables hybrid connectivity and dynamic resource allocation, effectively addressing urban network congestion and variable service demands.

Simulation results based on a realistic Manhattan grid urban model demonstrate that the proposed architecture can achieve up to 47% higher available bandwidth compared to conventional approaches, while reliably meeting stringent service requirements under high-density traffic conditions of 500 EVs/km^2^. The system exhibits strong congestion control by intelligently offloading traffic to aerial nodes, and its computational efficiency is supported by a polynomial-time complexity profile, ensuring scalable, real-time operation through optimized signaling protocols that align with existing V2X standards.

Application-level performance optimization is achieved through three core mechanisms: bandwidth relaying, context-aware allocation, and proactive video delivery. While the importance of interference—specifically co-channel and adjacent-channel effects—is acknowledged, the current version does not include their detailed modeling or mitigation. Instead, interference management is identified as a key focus for future development.

While the proposed system includes hybrid FSO/RF fallback mechanisms to maintain link availability, quantification of FSO degradation under weather events such as fog or rain remains an open challenge. Future simulations will incorporate empirical weather models to characterize FSO reliability under various atmospheric conditions, supporting more robust fallback decisions and throughput prediction.

Similarly, drone endurance and energy consumption are recognized as constraints on sustained aerial coverage. The current analysis does not yet include power or battery models, which may affect long-term feasibility. Future work will model UAV power budgets, flight time limitations, and dynamic deployment costs to evaluate operational viability, including energy-aware scheduling and cost–performance trade-offs for large-scale deployments.

Subsequent research will include the design and evaluation of an interference mitigation framework, incorporating dynamic spectrum allocation, adaptive power control, and standardized channel models such as 3GPP UMi and ITU-R HAPS. These mechanisms will support more effective handling of cross-layer interference in congested conditions. Performance evaluation will target key metrics such as signal-to-interference-plus-noise ratio (SINR), system outage probability, and handover reliability between aerial and ground segments.

Future work will also enhance aerial resource deployment through energy-efficient drone operations and cost-optimized HAP utilization, expanding service coverage and sustainability. End-to-end validation using 6G-capable prototypes will provide real-world insights into sustained throughput, latency, and seamless handovers under peak load conditions. Additionally, coordinated channel access and other interference-aware protocols will be explored to improve stability across hybrid aerial–vehicular links.

The architecture’s inherent multi-layer structure and distributed resource management provide a strong foundation for low-latency, resilient service delivery in urban mobility environments. Planned enhancements to user-perceived quality mechanisms and interference resilience will support the transition from conceptual framework to practical deployment, maintaining its advantages in bandwidth provisioning, congestion resistance, and multimedia service continuity.

## Figures and Tables

**Table 1:** Summary of related work and limitations.

Ref.	Focus/Contribution	Technology	Strength	Limitation
[1]	Drone–vehicle collaboration for improved coverage and reliability	Drone-assisted V2X	Enhances connectivity, supports mobility	Lacks support for adaptive video delivery
[2,3,4]	Challenges of network instability in urban vehicular environments	Cellular vehicular networks	Highlights QoS degradation factors	No mitigation strategy for infotainment traffic
[5,6]	Analyze 5G limitations in high-density, high-mobility conditions	5G cellular, mmWave	Shows performance bottlenecks	Lacks scalability and robustness for infotainment
[7,8]	Introduction of 6G technologies for enhanced capacity and latency	Terahertz (THz), mMIMO	Promises significant gains in capacity	Underexplored in vehicular infotainment scenarios
[9]	Drones in smart transportation systems	Drone-assisted V2X	Flexible deployment, rural coverage	Static, lacks media awareness or adaptability
[10]	SDN and RL for drone-assisted edge computing	SDN, MEC, reinforcement learning	Efficient task offloading	Ignores video streaming and UAV–media coordination
[11]	Multi-drone learning for responsive network control	Distributed learning, multi-UAV	Enhances real-time adaptation	No support for video layer control
[12]	Spectrum sharing for drone–ground coexistence	Hybrid licensed/unlicensed bands	Improved PHY coexistence	Lacks scalable content delivery framework
[13,14,15,16]	HAPs for wide-area and persistent connectivity	HAPs (balloons, solar drones)	Low latency, wide coverage	No content-layer integration for infotainment
[17,18]	High-speed optical links between aerial platforms	FSO links	High data rate, interference-free	Weather-sensitive, ground-level use not addressed
[19]	Hybrid switching to overcome FSO reliability issues	FSO/RF hybrid	Resilient against weather interference	Focused on inter-HAP, not UAV–ground applications
[20,21,22]	SVC for layered video compression and transmission	H.264/H.265/H.266	Improved compression and scalability	Lacks dynamic integration with network state
[23]	Adaptive video layer delivery	SVC	Flexible bandwidth usage	Not integrated with UAV or mobility systems
[24]	Tile-based 360° video delivery	Tiling, viewport-aware streaming	Reduces redundancy	No UAV or context-aware delivery support
[25]	Joint segment and frame bitrate adaptation	Bitrate adaptation	Improves QoE with fine-grained control	High computational complexity
[26]	Dynamic bitrate adaptation and bandwidth allocation	Adaptive streaming	Efficient resource utilization	Limited scalability in dynamic networks
[27]	VLC for short-range V2V communication	VLC	High-speed local data exchange	No integration with aerial relays or global delivery

**Table 2:** Transmission distances and capacity of wireless communication technologies.

Wireless Communication Technology	Maximum Communication Distance	Maximum Capacity
mmWave	100 m	20 Gbps
THz	500 m	240 Gbps
FSO	HAPs 20 km height	335 Gbps
VLC	14 m	270 Mbps

**Table 3:** Expected bandwidth requirements for 2D and 360-degree videos.

Video Classification	Resolution and Frame Rate Specifications	Expected Bandwidth
	7680 × 4320 60 fps	135–156 Mbps
2D video	3840 × 2160 60 fps	38–44 Mbps
	2560 × 1440 60 fps	17 Mbps
	1280 × 720 60 fps	5 Mbps
	7680 × 3840 60 fps	125–309 Mbps
360-degree video	4090 × 4090 60 fps	70–174 Mbps
	2890 × 1920 60 fps	33–82 Mbps

**Table 4:** Computational complexity characteristics of the proposed algorithm and the baseline.

Characteristic	This Work	[1]
Problem domain	Heuristic-driven video streaming and relay planning	Analytical coverage and sum-rate optimization
Dominant variables	*M*, *L*, *R*, *N*	*D* (channels or drones)
Worst-case complexity	O(M·logM+L·M+R·N)	$O(D^{2})$–$O(D^{3})$
Operations cost	Sorting, mapping, path planning	Closed-form formulas + iterative convex solves
Scalability	Linear/log-linear in practical parameters	Polynomial in *D*; suitable for moderate drone sets

## Data Availability

Simulation data may be shared with qualified academic researchers for collaborative purposes, subject to approval by the corresponding author and compliance with institutional data sharing agreements.
